# In situ architecture of the ER-mitochondria encounter structure

**DOI:** 10.1038/s41586-023-06050-3

**Published:** 2023-05-10

**Authors:** Michael R. Wozny, Andrea Di Luca, Dustin R. Morado, Andrea Picco, Rasha Khaddaj, Pablo Campomanes, Lazar Ivanović, Patrick C. Hoffmann, Elizabeth A. Miller, Stefano Vanni, Wanda Kukulski

**Affiliations:** 1 MRC Laboratory of Molecular Biology, Francis Crick Avenue, Cambridge CB2 0QH, UK; 2 Department of Biology, University of Fribourg, 1700 Fribourg, Switzerland; 3 SciLifeLab, Tomtebodavägen 23, 171 65 Solna, Sweden; 4 Department of Biochemistry, University of Geneva, Quai Ernest Ansermet 30, 1211 Geneva, Switzerland; 5 Institute of Biochemistry and Molecular Medicine, University of Bern, Bühlstrasse 28, 3012 Bern, Switzerland; 6 Graduate School for Cellular and Biomedical Sciences, University of Bern, Mittelstrasse 43, 3012 Bern, Switzerland; 7 Department of Anatomy and Cell Biology, McGill University, Montreal, H3A 0C7, Canada; 8 Max Planck Institute of Biochemistry, 82152 Martinsried, Germany; 9 Max Planck Institute of Biophysics, 60438 Frankfurt am Main, Germany

## Abstract

The endoplasmic reticulum and mitochondria are main hubs of eukaryotic membrane biogenesis that rely on lipid exchange via membrane contact sites^1–3^, but the underpinning mechanisms remain poorly understood. In yeast, tethering and lipid transfer between the two organelles is mediated by the ER-mitochondria encounter structure (ERMES), a four-subunit complex of unclear stoichiometry and architecture^4–6^. Here we determined the molecular organization of ERMES within cells using integrative structural biology by combining quantitative live-imaging, cryo-correlative microscopy, subtomogram averaging and molecular modelling. We found that ERMES assembles into approximately 25 discrete bridge-like complexes distributed irregularly across a contact site. Each bridge consists of three synaptotagmin-like mitochondrial lipid binding protein (SMP) domains oriented in a zig-zag arrangement. Our molecular model of ERMES reveals a pathway for lipids. These findings resolve the *in situ* supramolecular architecture of a major interorganelle lipid transfer machinery and provide a basis for mechanistic understanding of lipid fluxes in eukaryotic cells.

Lipids are fundamental constituents of eukaryotic cells and are mainly synthesized in the endoplasmic reticulum (ER). The ER supplies lipids to other organelles for membrane homeostasis and expansion. Although lipids and proteins traffic via vesicles from the ER to outlying organelles, the bulk of cellular lipid flux occurs through membrane contact sites (MCS)^7^, which are regions where two organelles are physically apposed^8^. For lipid flux between the ER and mitochondria, MCS are the only conduit^1–3^. In yeast, ER-mitochondria MCS are mediated by the ER-mitochondria encounter structure (ERMES), which is necessary for respiratory function as well as maintenance of both the mitochondrial genome and mitochondrial morphology^4,9^. ERMES mediates the transport of phospholipids between ER and mitochondria^5,6^, likely underpinning its roles in mitochondrial function. ERMES is composed of four core components, Mmm1, Mdm12, Mdm34 and Mdm10, which have molecular weights between 31 and 56 kDa^4^. Three components (Mmm1, Mdm12 and Mdm34) share conserved synaptotagmin-like mitochondrial-lipid-binding (SMP) domains, characteristic of many MCS lipid transfer proteins in yeast and mammals^10–12^. Although structural information on SMP domains is available, including for Mmm1 and Mdm12, it remains unknown how SMP domains arrange between two organelles to drive inter-organelle lipid transfer^13–17^. Specifically, whether ERMES subunits arrange into stoichiometric complexes that form lipid shuttles or continuous conduits is unclear.

## Number of ERMES molecules per MCS

**Extended Data Figure 1:**
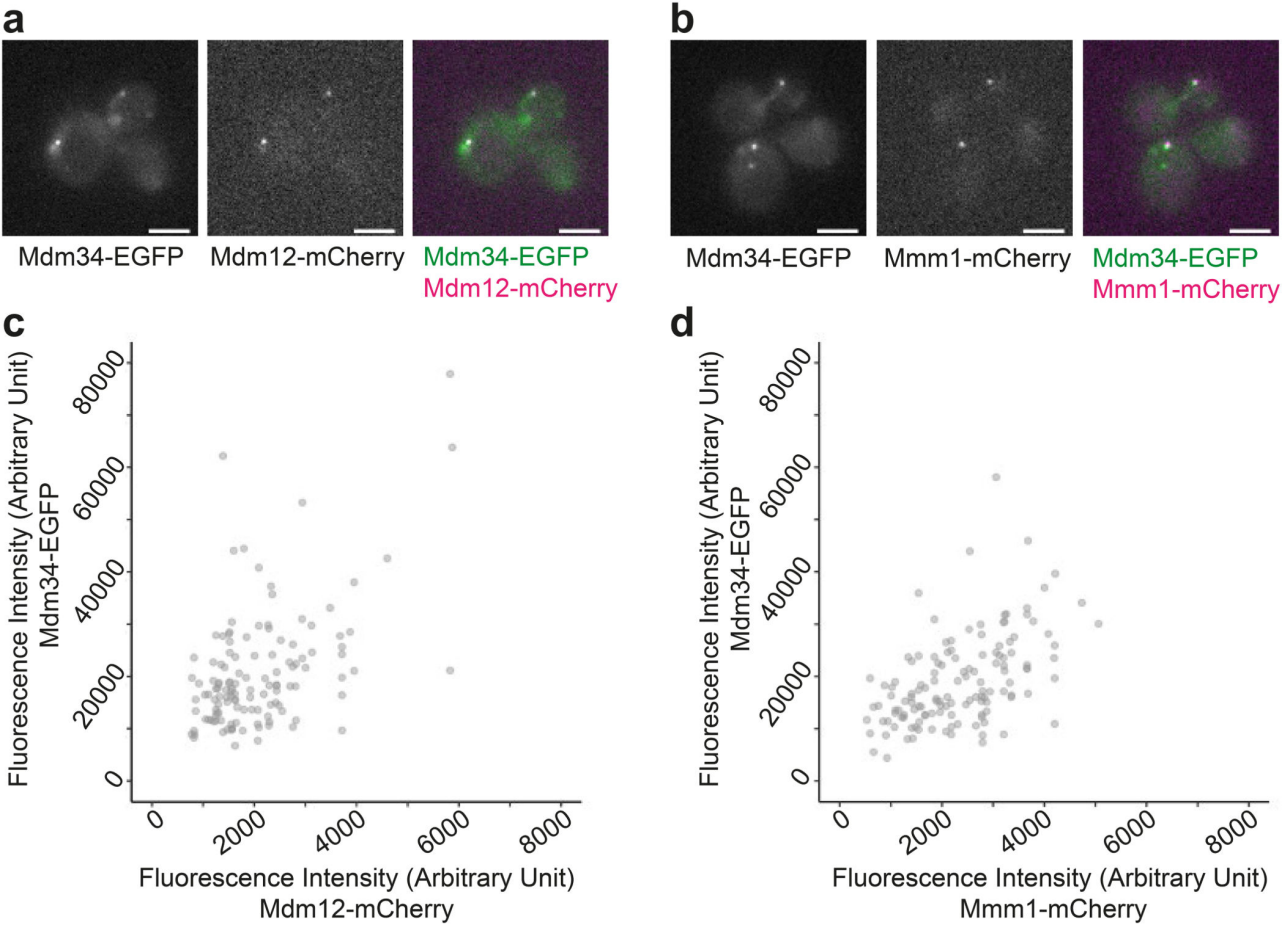
Correlation between simultaneously labelled ERMES components. **a:** Fluorescence live cell microscopy of cells expressing Mdm34-EGFP and Mdm12-mCherry. Individual channels and merge are shown. **b:** Fluorescence live cell microscopy of cells expressing Mdm34-EGFP and Mmm1-mCherry. Individual channels and merge are shown. **c:** The fluorescence intensity of Mdm34-EGFP and Mdm12-mCherry, labelled in the same cells, correlates in puncta. N=128 puncta, Pearson’s correlation R=0.566, p=3.38*10^-12^ (two-sided). **d:** The fluorescence intensity in a.u. of Mdm34-EGFP and Mmm1-mCherry, labelled in the same cells, correlates in puncta. N=134 puncta, Pearson’s correlation R=0.529, p=5.18*10^-11^ (two-sided). Scale bars are 3 μm.

**Figure 1:**
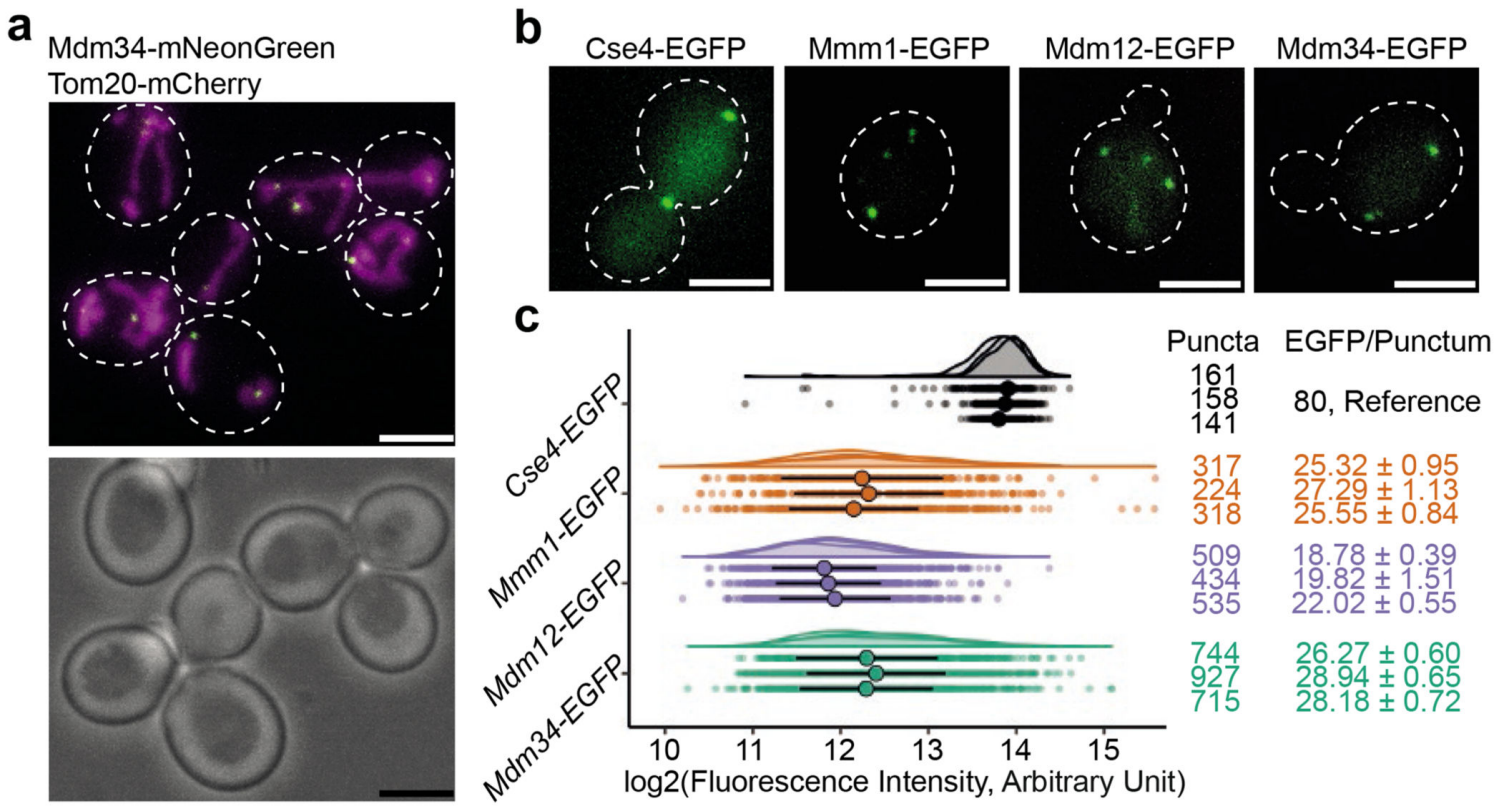
The number of molecules of ERMES components per MCS. **a:** Live cell imaging of budding yeast cells expressing Tom20-mCherry (magenta), marking mitochondria, and Mdm34-mNeonGreen (green), marking ERMES-mediated MCS. White dashed outlines mark cell boundaries in the fluorescence image (top) according to bright field image (bottom). **b:** Live cell FM of yeast cells expressing either Cse4-EGFP, Mmm1-EGFP, Mdm12-EGFP or Mdm34-EGFP, forming diffraction limited puncta. White dashed outlines mark cell boundaries. Cells expressing the kinetochore protein Cse4-EGFP, of which the number of molecules per diffraction limited spot is known^36^ were used as a reference to determine the number of molecules of ERMES components. **c:** Fluorescence intensity quantifications of puncta of EGFP-tagged Cse4 (grey), Mmm1 (orange), Mdm12 (purple) and Mdm34 (green), represented as dot plots as well as half-violin plots. Three experimental repeats are shown. Each data point is one punctum. Large points represent median and vertical lines MAD, for each experimental repeat. Using Cse4-EGFP as reference, median +/-MAD fluorescence intensities were transformed into median +/-MAD numbers of EGFP molecules/punctum (right column). Left column indicates number of analysed puncta (N). Scale bars are 3 μm.

We used an integrative structural biology approach to reveal the supramolecular structure of ERMES-mediated MCS in budding yeast, determine the *in situ* organization of ERMES SMP domains and obtain insights into the mechanism of lipid transfer. When observed by fluorescence microscopy (FM), the three SMP-domain containing ERMES components Mmm1, Mdm12 and Mdm34 organise as diffraction limited puncta that correspond to ER-mitochondria MCS^4^ (Fig. 1a). We first determined the number of SMP-domain containing ERMES components per puncta and their ratio by quantitative live cell FM^18,19^. Fluorescence intensity measurements provided us with an estimate of the median absolute number of molecules per MCS of the three ERMES components (Fig. 1b). On average, the median number of Mmm1 and Mdm34 molecules matched closely (mean of medians=26.1, standard deviation (SD) ±1.1, and 27.8, SD ±1.4, molecules/punctum, respectively), and was also similar for Mdm12 (mean of medians=20.2, SD ±1.7, molecules/punctum) (N=3 experimental repeats, Fig. 1c). When we imaged two simultaneously labelled ERMES components, we found that their fluorescence intensities were correlated in the puncta (Extended Data Fig. 1, Pearson’s correlation P<0.001 for both Mdm34-EGFP with Mdm12-mCherry, and Mdm34-EGFP with Mmm1-mCherry), indicating that the number of molecules of one component conforms to the number of molecules of the other component within individual MCS. We thus propose that ER-mitochondria MCS consist of similar protein copy numbers of the three SMP-domain containing ERMES components. The lower abundance of Mdm12 could reflect its assembly properties, as Mdm12 is cytosolic and its targeting to MCS might require its interaction partners Mdm34 and Mmm1 to be present^14,20^. In contrast, Mmm1 is anchored to the ER by a transmembrane domain, and Mdm34 associates tightly with the outer mitochondrial membrane (OMM) protein Mdm10^20,21^. Nevertheless, *in vitro* the SMP domains of Mmm1 and Mdm12 form a stable equimolar complex^16^. Thus, taken together, our findings support a model of ERMES assembling as a protein complex with equimolar stoichiometry between Mmm1, Mdm12 and Mdm34.

## ERMES forms bridge structures

**Extended Data Figure 2:**
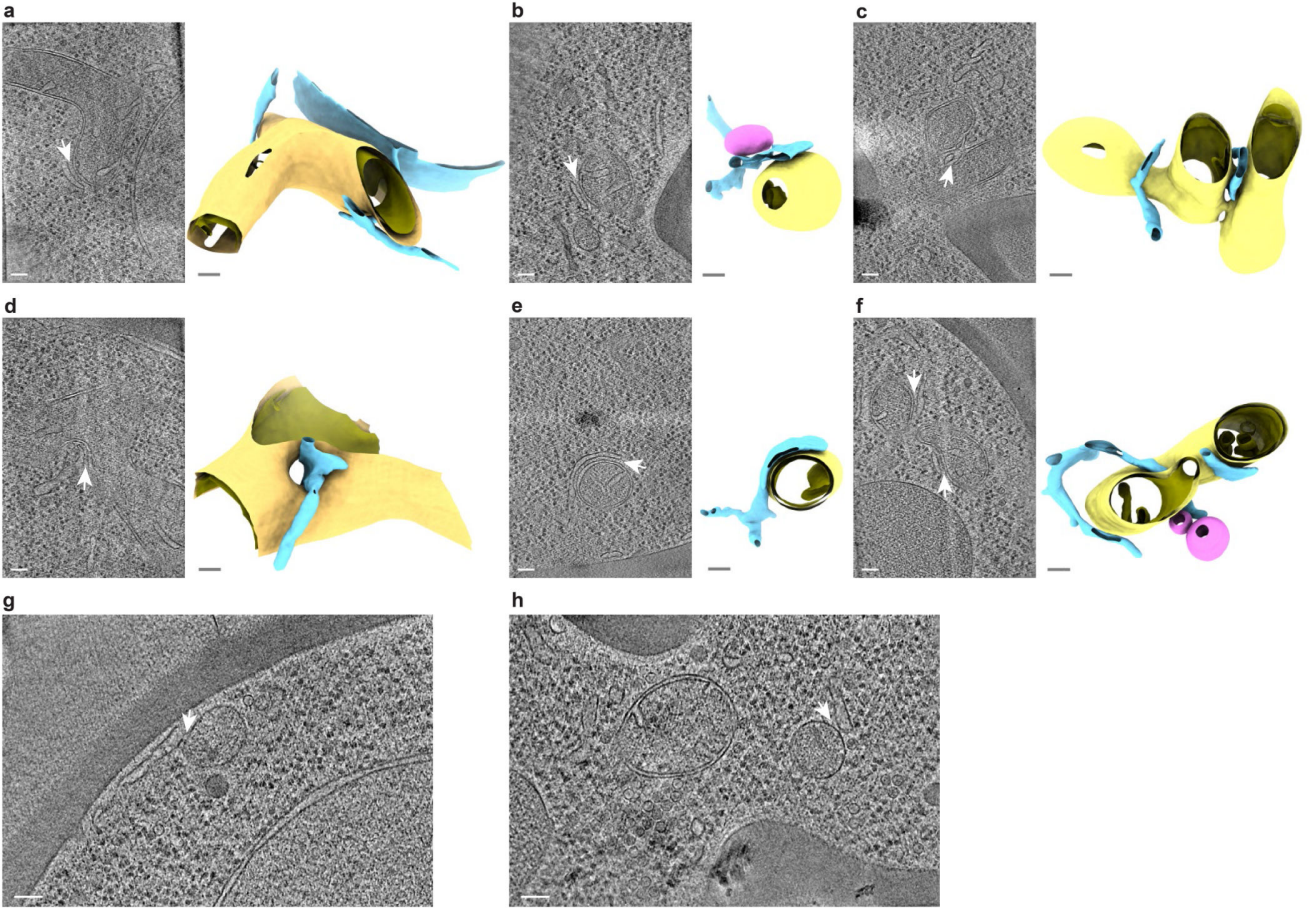
MCS imaged by cryo-ET at Mdm34-mNeonGreen signals. **a-f:** Diversity of membrane morphologies of ER-mitochondria MCS imaged by cryo-ET at locations of Mdm34-mNeonGreen. Six representative examples from a data set of 51 tomograms are shown. Left panels are virtual slices through electron cryo-tomograms. ER-mitochondria MCS are indicated by white arrows. Right panels are segmentation models of the ER (blue), the OMM (yellow), the IMM (mustard) and peroxisomes (pink). The segmentation models are rotated relative to the virtual slices to better visualize the MCS. The example in e is the same as shown in Fig. 3e. g, h: ER-peroxisome contacts imaged by cryo-ET at locations of Mdm34-mNeonGreen. Approximately 15% of Mdm34-mNeonGreen puncta contained ER-peroxisome MCS (indicated by white arrows) rather than ER-mitochondria MCS. Peroxisomes are identified as spheroid single-membrane vesicles more than 100 nm in diameter with dense interior^77,78^. Scale bars are 100 nm. Note that due to the perspective view, scale bars in the panels showing segmentation models apply only to the front plane of the scenes.

**Figure 2:**
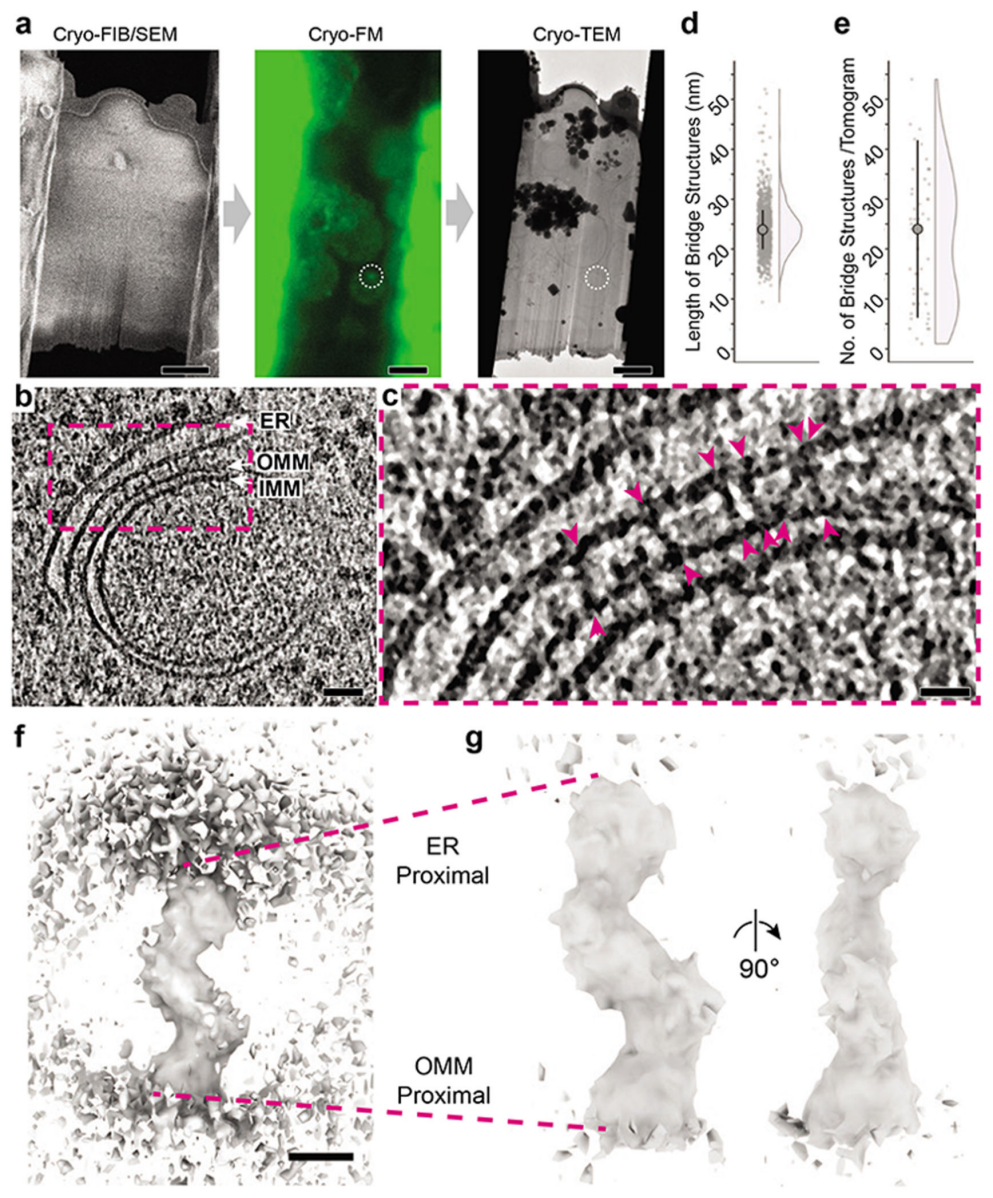
ERMES-mediated MCS consist of bridge structures connecting the two membranes. **a:** The cryo-CLEM workflow includes three microscope steps. Thinning of cells into lamellae by cryo-FIB milling visualized by scanning EM (SEM); cryo-FM of lamellae to localize Mdm34-mNeonGreen marked ERMES puncta (white dashed circle); and cryotransmission EM (TEM) for acquisition of electron cryo-tomograms. **b:** Virtual slice through an electron cryo-tomogram acquired at an Mdm34-mNeonGreen punctum, showing an ER-mitochondrial MCS. The ER, OMM and IMM are indicated. **c:** Zoom into the region in dashed pink box in b. The arrowheads indicate bridge-like connections between the ER and the OMM. **d:** The length of the bridge structures in nm. Dot plot and half-violin plot. Large point indicates mean (24.2 nm, N=1098 bridges from 51 tomograms), vertical lines SD. **e:** The number of bridge structures found per electron cryo-tomogram acquired at Mdm34-mNeonGreen puncta. Dot plot and half-violin plot. Large point indicates median (24, N=51 tomograms), vertical lines MAD. **f:** 3D map of the bridge structure obtained by STA. The ER (top) and OMM (bottom) membranes are partially visible. **g:** STA map at higher contour level than in f, therefore membranes are not visible. Two views rotated by 90° along the major axis. The map represents the cytosolic portion of the bridge, with regions proximal to the ER and OMM indicated. Scale bars are 3 pm in a, 50 nm in b, 20 nm in c, and 5 nm in f Note that due to the perspective view, the scale bar in f only applies to the front plane of the scene.

A stoichiometric ERMES complex is compatible with two proposed functional models: that of a stable conduit bridging the ER and mitochondria, or of a mobile shuttle moving between these two organelles. To determine the mode of lipid transport at ER-mitochondria MCS, we visualized ERMES MCS in vitrified yeast cells using correlative light and electron cryo microscopy (cryo-CLEM). Vitrified cells were thinned into lamellae using cryo-focused ion beam (FIB) milling^22^, then imaged using cryo-FM to determine the presence and position of ERMES puncta, marked by Mdm34-mNeonGreen, within lamellae (Fig. 2a). Regions of lamellae with ERMES puncta were imaged using electron cryo-tomography (cryo-ET). This approach reliably directed data collection to ER-mitochondria MCS. These MCS consisted of diverse morphologies of ER and mitochondria (Extended Data Fig. 2a-f). ER membranes in contact with mitochondria did not show preferred curvature: convex (29%), concave (32%), convex and concave in different locations of the same MCS (6%), or nearly flat membranes (33%, N=63 MCS) were all observed. In some cases, the ER was wrapped around the mitochondrial surface (Extended Data Figs. 2a, e); in others, ER tubules passed through holes in mitochondria (Extended Data Figs. 2d, f). Occasionally, we found peroxisomes near ER-mitochondria MCS, often in close contact with the ER (Extended Data Figs. 2b, f). Notably, in addition to 51 tomograms of ER-mitochondria MCS, 9 tomograms collected at fluorescent ERMES puncta contained no ER-mitochondria MCS but instead ER-peroxisome MCS (Extended Data Fig. 2g, h). These observations are in line with previous findings that ERMES can localize to MCS involving peroxisomes^23,24^.

Importantly, the cryo-CLEM approach provided us with confidence that the imaged MCS contained a significant amount of ERMES (Fig. 2b). Within ER-mitochondria MCS we found numerous dense, bridge-like structures spanning the gap between the two organelles (Fig. 2c). These structures spanned on average a length of 24.2 nm (SD=4.76 nm, N=1098 bridges, Fig. 2d). A median of 24 bridge structures were observed per tomogram (median absolute deviation (MAD)=17.8, N=51 tomograms) (Fig. 2e). The number of bridge structures is in close agreement with the number of molecules of Mmm1, Mdm12 and Mdm34 per diffraction limited puncta. These findings suggest that the majority of bridge structures, which are located at puncta of Mdm34-mNeonGreen, represent the ERMES complex forming a continuous structure between the ER and mitochondrial membranes.

**Extended Data Figure 3:**
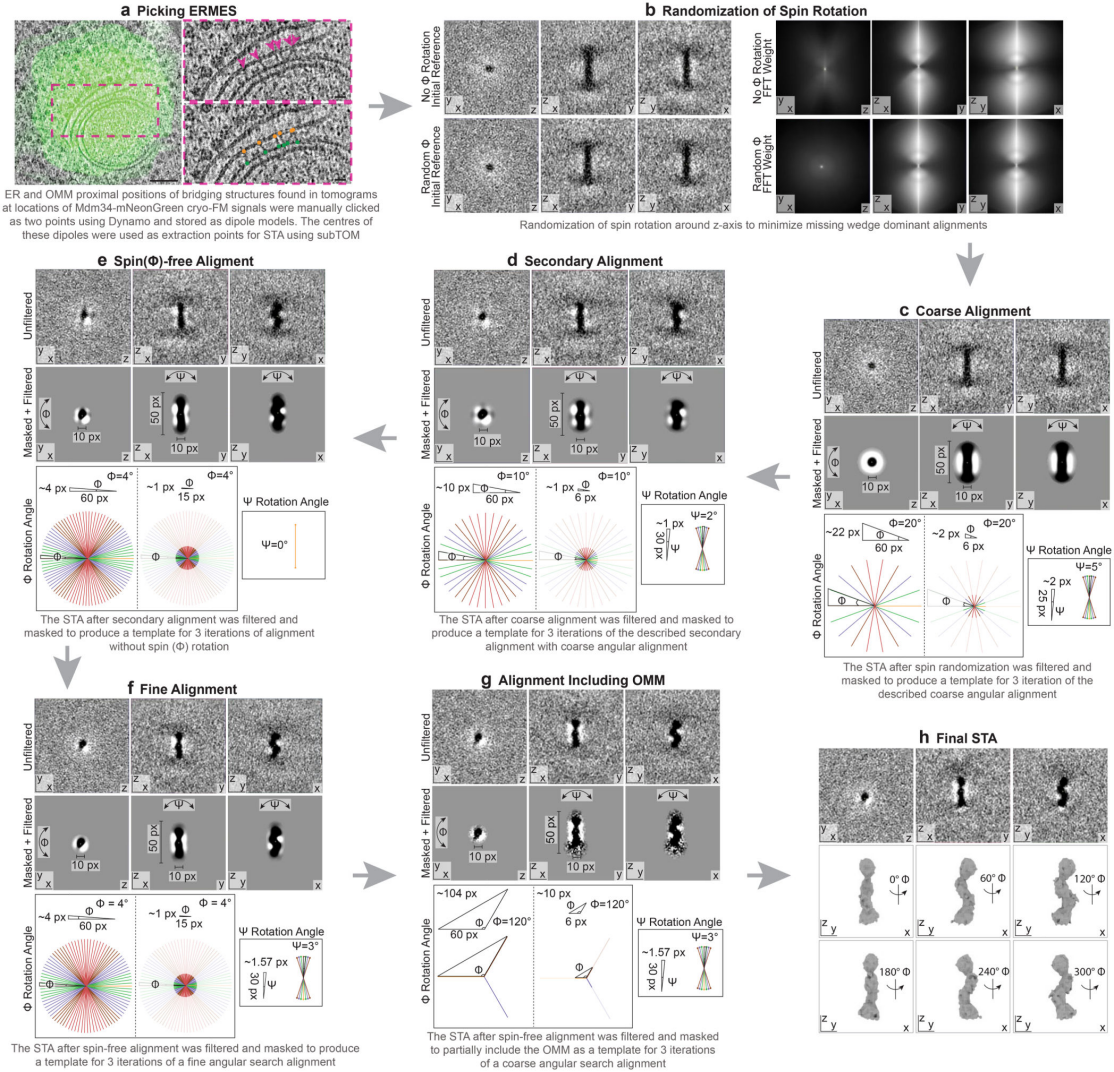
Subtomogram averaging pipeline. The strategy using sequential alignment steps is outlined in steps a - h. The data set consisted of manually picked coordinates of the ER and OMM anchor points of 1098 bridge structures from 51 electron cryo-tomograms at positions of Mdm34-mNeonGreen signals. The resolution of the final STA map is potentially affected by a minor fraction of bridge-like particles of different identity, which could contribute noise. Images in a are the same as shown in Fig. 2b and c, with modifications to the overlay. Scale bars in a are 100 nm (left image) and 20 nm (images in pink dashed boxes).

**Extended Data Figure 4:**
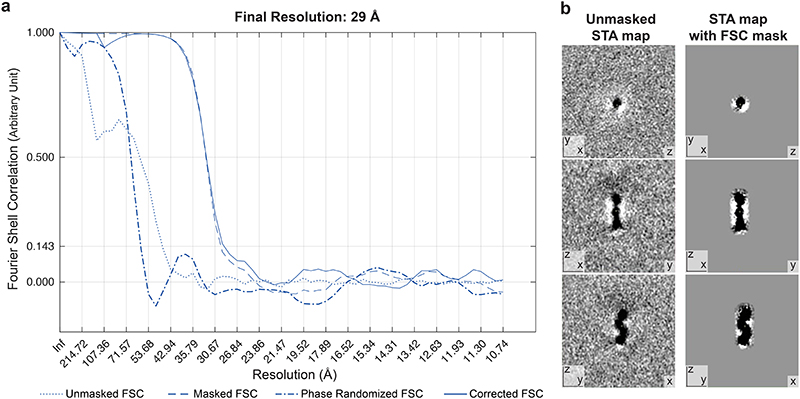
Resolution estimate of STA map. **a:** Fourier shell correlation, calculated according to^59^. The final resolution corresponds to FSC=0.143. **b:** Left panel: Three virtual slices through the unmasked STA map. Right panel: Three virtual slices through the STA map masked for FCS calculation.

**Extended Data Figure 5:**
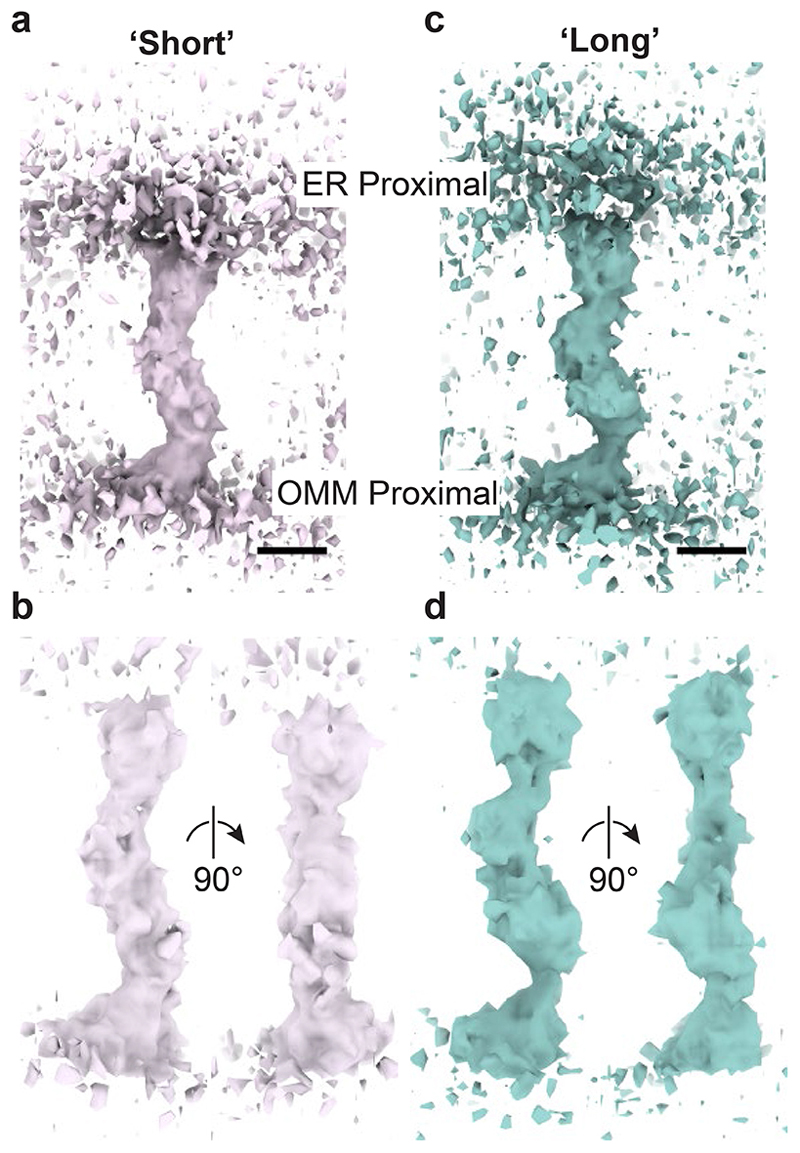
STA maps of short and long bridges. **a, b:** STA map of short class (pink). **c, d:** STA map of long class (teal). The two classes were obtained by halving the data set of 1098 bridge structures according to bridge length and subjecting both classes separately to the procedure shown in Extended Data Fig. 3. In a and c the maps are shown at lower contour levels than in b and d. Scale bars are 5 nm. Note that due to the perspective view, scale bars apply only to the front plane of the scenes.

To gain structural details of the bridges and insight into the organization of ERMES, we used subtomogram averaging (STA) to determine an average 3D map from subvolumes containing the bridge structures (Extended Data Figs. 3, 4, Extended Data Table 1). The resulting map fills the distance between the ER and mitochondria, likely corresponding to the full cytosolic portion of the ERMES complex (Fig. 2f). There are two bends along the map which produce a zig-zag arrangement of three segments similar in size and shape (Fig. 2g, Supplementary Video1). To assess how bridges of different length (Fig. 2d) differ in structure, we separated the bridges according to the length they spanned between the membranes into two equal groups, short (max=23.9 nm, min=9.4 nm, median=21.3 nm, MAD=2.3 nm, N=549) and long bridges (max=52.0 nm, min=23.9 nm, median=26.6 nm, MAD=2.6 nm, N=549). We then subjected these two classes to STA. While the resulting STA maps were overall similar, the difference in length was mostly attributable to the bridge segments close to OMM and ER membrane. In the STA of the long class, these segments appear straighter and attenuated at the connections to the central segment (Extended Data Fig. 5). These differences indicate a certain degree of flexibility in the zig-zag arrangement that appears to modulate bridging length.

The overall bridge structure suggests the assembly of three SMP domains arranged consecutively in a continuous string. The order of the arrangement is deducible from available information on the three SMP domain containing components: Mmm1 is anchored in the ER and interacts with Mdm12; Mdm34 interacts with Mdm12 and with the OMM protein Mdm10^16,20,21^. Thus, in our map, the segment nearest the ER is likely to contain the Mmm1 SMP domain, the central segment Mdm12, and the segment nearest the OMM Mdm34. Such an organization fits with the equimolar ratio of components determined by FM. We propose that ERMES can bridge the space between the ER and mitochondria through a stable complex which is anchored to both membranes simultaneously. In this complex, each component contributes one SMP domain, strung in a zig-zag assemblage that can adjust to adopt different bridging lengths.

## Organization of bridges within MCS

**Figure 3:**
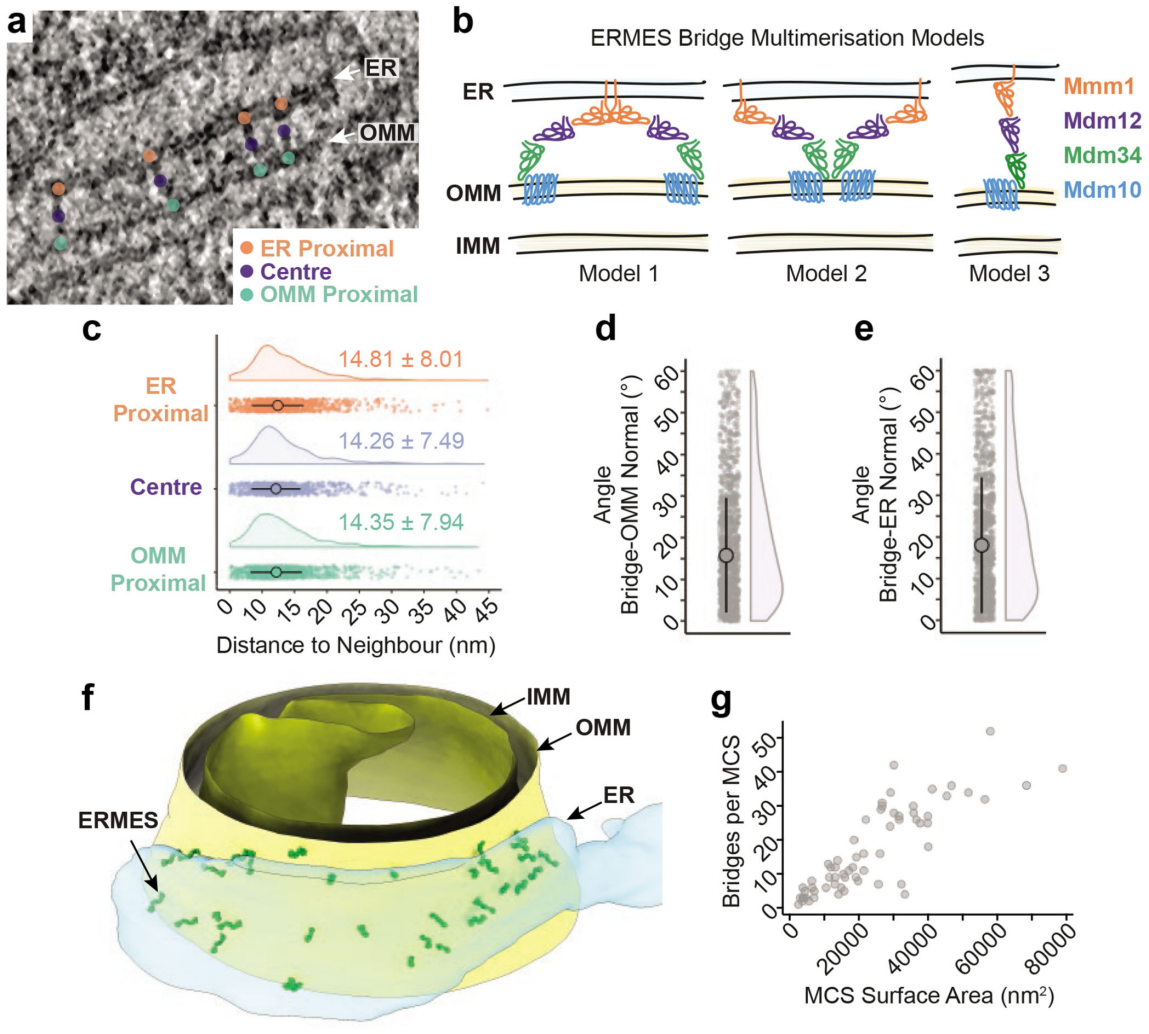
Supramolecular organization of ERMES within MCS. **a** Coordinates of ER membrane anchor points (orange), centre points (purple) and OMM anchor points (green) in electron cryo-tomograms. **b:** Three possible models of how ERMES bridges could be arranged relative to each other, consistent with the STA map. Model 1: ERMES dimerizes via Mmml as observed *in vitro^14,16^;* ER anchor points of neighbouring bridges would be close to each other. Model 2: ERMES dimerizes via Mdm34^14^ or Mdm10; OMM anchor points of neighbouring bridges would be close to each other. Model 3: Neither model 1 nor 2 applies if neighbouring bridges are similarly close at the ER, the bridge centre and the OMM. **c:** Dot plot and half-violin plot of the distances between nearest neighbouring bridges, measured between the ER anchor points (orange), the bridge centres (purple), and the OMM anchor points (green), respectively. Large point indicate medians, vertical lines MAD, both also given as numerical values (N=1095 bridges from 49 tomograms) **d:** The angle by which each bridge is tilted relative to the OMM normal. Dot plot and half-violin plot. Large point indicates median, vertical lines MAD (N=1098 bridges from 51 tomograms). **e:** The angle by which each bridge is tilted relative to the ER membrane normal. Dot plot and half-violin plot. Large point indicates median, vertical lines MAD (N=1098 bridges from 51 tomograms). **f:** Segmentation model of an electron cryo-tomogram, showing the distribution of ERMES bridge structures within MCS. The STA map was placed at the positions of individual bridge structures, indicated as ERMES. OMM, IMM and ER are also indicated. **g:** The number of bridges per MCS, plotted as a function of the surface area of the ER membrane in contact with the OMM (N=63 MCS from 49 tomograms).

*In vitro*, the Mmm1 and Mdm34 SMP domains homo-dimerize like SMP domains of other lipid transfer proteins and could thus mediate dimerization of ERMES bridges through an interaction near the ER or the OMM membrane, respectively^13–16,25^. Since our STA approach averaged individual bridges, we could have missed dimerized bridges. We therefore used the tomographic coordinates of bridge-membrane anchor points on the ER and the OMM, as well as the centre points of the bridges, to determine whether the bridges had a specific arrangement relative to each other (Fig. 3a). For each point, we measured the distance to its nearest neighbouring point. We reasoned that if ERMES dimerized via its ER or OMM-associated components, the distance between the respective anchor points of two neighbouring bridges would be short. Furthermore, if dimerization occurred preferentially near one of the two membranes, the distance between the respective anchor points would be closer than that between anchor points on the other membrane or between centre points (Fig. 3b). Our analysis revealed that for ER anchors, bridge centre points and OMM anchors, the distances between nearest neighbours were equivalent (median +/-MAD: 14.8 nm +/-8.0 nm, 14.3 nm +/-7.5 nm and 14.3 nm +/-7.9 nm, respectively; Fig. 3c). For each type of anchor point, a fraction of bridges with very close neighbours could be found, indicating that some bridges might associate with neighbouring bridges, possibly forming dimers. However, the distances separating most anchor points indicate no direct association of neighbouring bridges. Thus, the majority of the bridges do not form an arrangement involving homodimerization of Mmm1 or Mdm34 SMP domains.

**Extended Data Figure 6:**
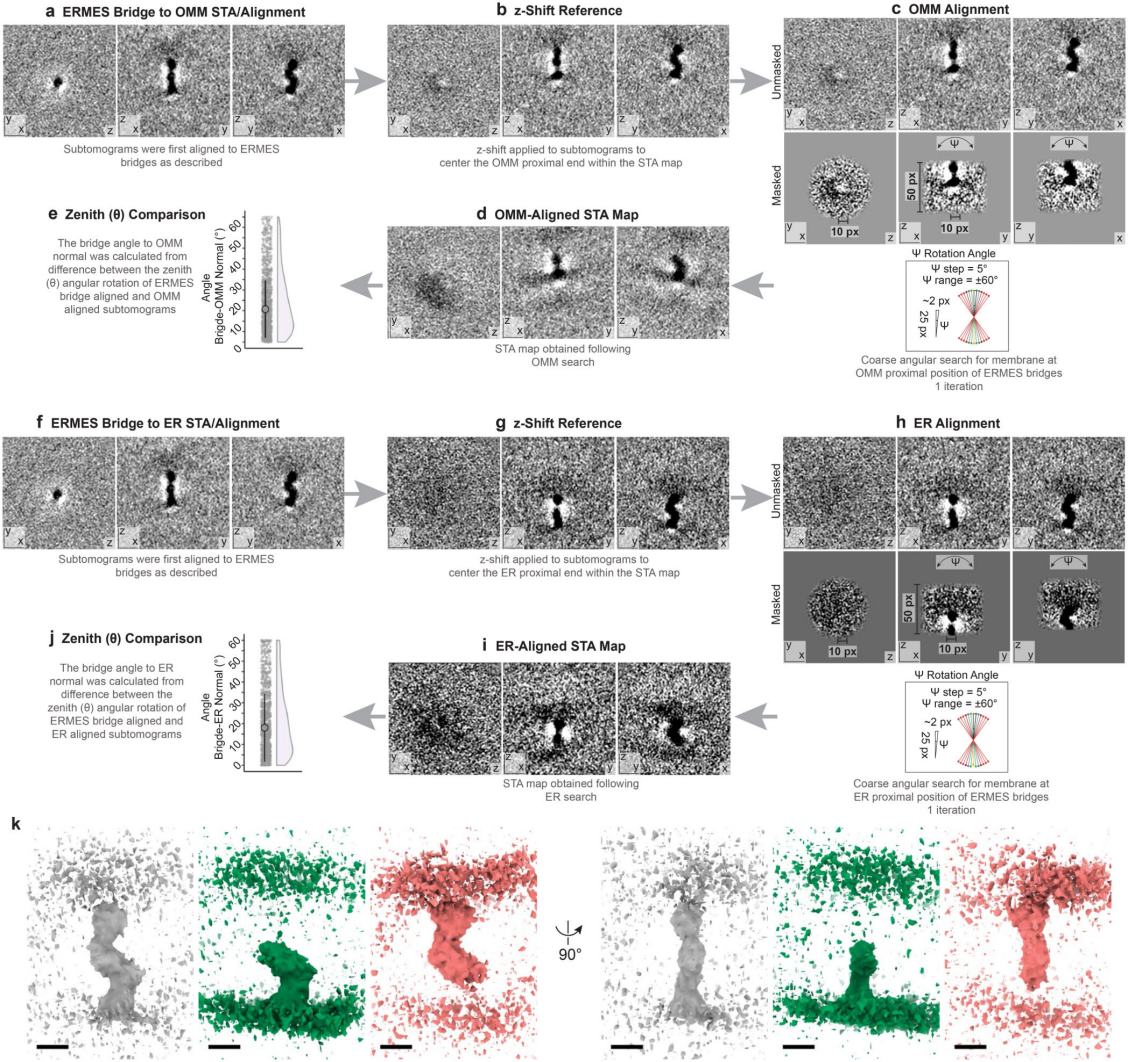
Orientation of the bridges relative to the membranes. **a-d:** STA alignment strategy to determine the angle between the bridges and the OMM. **e:** This graph is the same as shown in Figure 3d. Large point indicates median, vertical lines MAD. N=1098 bridges from 51 tomograms. **f-i:** STA alignment strategy to determine the angle between the bridges and the ER. **j:** This graph is the same as shown in Figure 3e. Large point indicates median, vertical lines MAD. N=1098 bridges from 51 tomograms. **k:** Comparison of the STA maps obtained from different alignments. Grey: Full STA map obtained from the alignment strategy depicted in Extended Data Fig. 4. The STA map is the same as shown in Fig 2f and g, displayed at different contour level. Green: STA map obtained from the alignment strategy used to determine the angle between bridges and OMM, as depicted in panels a-d. Red: STA map obtained from the alignment strategy used to determine the angle between bridges and ER, as depicted in panels f-i. Scale bars are 5 nm. Note that due to the perspective view, scale bars apply only to the front plane of the scenes.

We did not include the membranes in the early steps of our STA procedure (Extended Data Fig. 3b-f) to avoid the membranes dominating the alignment of subvolumes containing the bridges. We noticed, however, that many bridges were not perpendicular to the membranes, but displayed an angled orientation. To assess this observation quantitatively, we performed two additional alignments of the previously aligned bridge subvolumes, now using the OMM or the ER as our target feature (Extended Data Fig. 6). The difference in the angle relative to the membranes between the full bridge alignment (Fig. 2f, g) and the OMM alignment position was 15.8° +/-13.8° (median +/-MAD, Fig. 3d). For the ER alignment position, this difference was 18.0° +/-16.3° (median +/-MAD, Fig. 3e). These results indicate that there is flexibility in the positioning of ERMES bridges relative to the membranes; possibly provided by hinge-like regions at the interfaces between the cytosolic and membrane parts of ERMES (Extended Data Fig. 6k). This flexibility in arrangement could help ERMES bridges accommodate varying membrane curvatures of the ER and the OMM as well as potential pushing and pulling forces acting on the organelles.

**Extended Data Figure 7:**
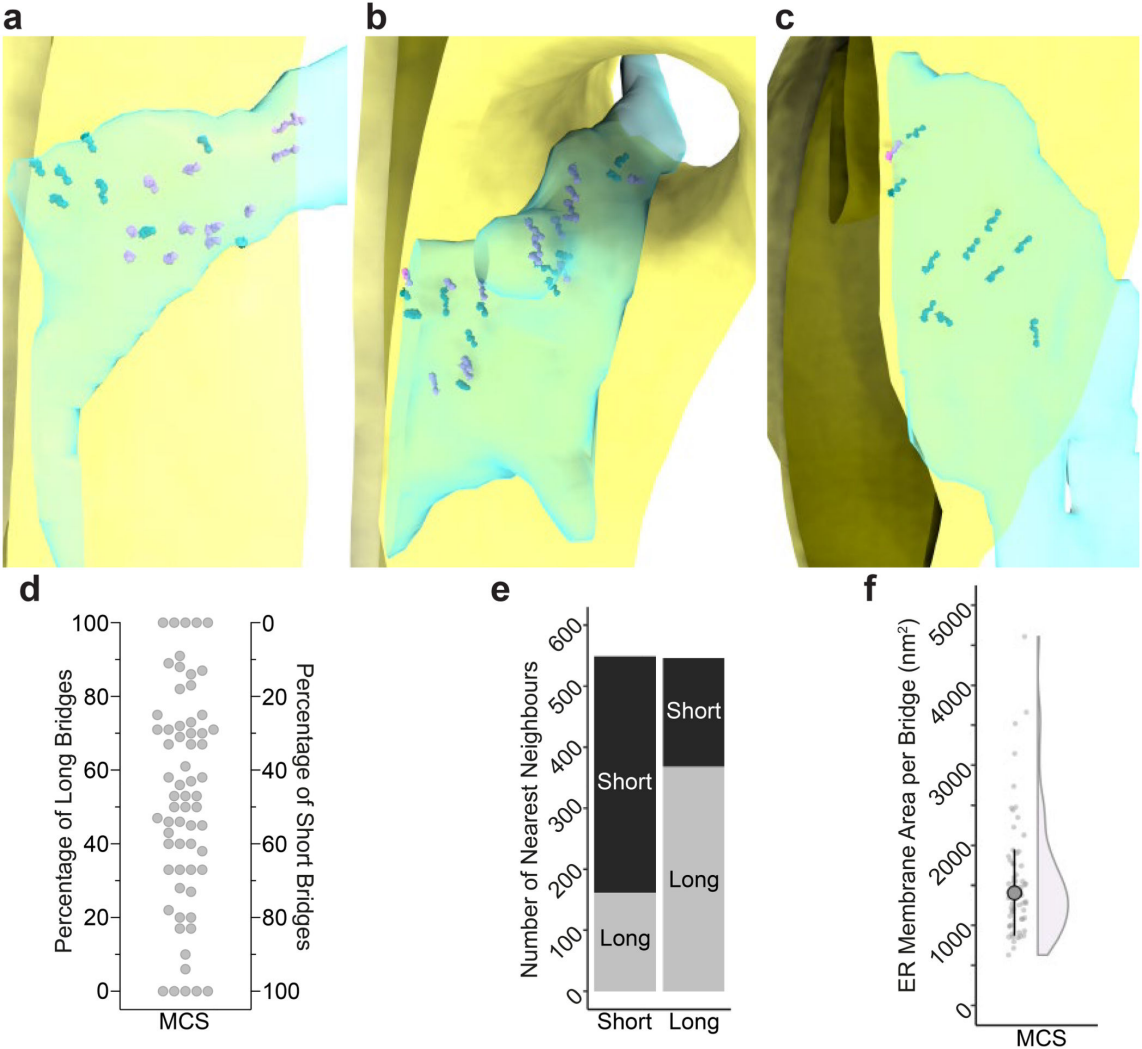
The distribution of bridges within the MCS ultrastructure. **a-c:** Segmentation models of MCS in three different tomograms, with STA maps of short (pink) and long (teal) classes placed back to the tomographic positions of individual bridges. OMM is in yellow, IMM in mustard, and ER in transparent light blue. **d:** Distribution of bridges of the long and short classes, across MCS. Each dot represents one MCS (N=64), plotted according to the percentage of long (left y-axis) and short bridges (right y-axis) it contains. **e:** Distribution of bridges of the long and short classes, within MCS. The number of short (left bar) and long (right bar) bridges that have a nearest neighbour belonging to the long (light grey bar fraction) and short (dark grey bar fraction) class. **f:** Dot plot and half-violin plot of the surface area of the ER membrane serviced by one ERMES bridge, determined per MCS. Large point indicates median, vertical lines indicate MAD. One outlier is not shown but included in median and MAD determination. N=63 MCS from 49 tomograms.

When we placed the STA map back into tomograms at positions of the individual subvolumes, we observed that the bridges were distributed within the MCS in clusters of varying density (Fig. 3f, Supplementary Videos 2–7). MCS contained various proportions of bridges of the short and long classes, showing no segregation into discrete MCS populations (Extended Data Fig. 7a-d). However, short and long bridges were more often nearest to another short (70%) or long bridge (67%), respectively (Pearson's Chi-square test, Chi-square=158.9, df=1, p-value<0.0001), indicating local clustering within MCS (Extended Data Fig. 7e). To estimate the area of membrane serviced by an individual ERMES bridge we measured the surface area of the ER membrane interfacing with the OMM. We found that one ERMES bridge occupied 1407 nm^2^ +/-540 nm^2^ (median +/-MAD, N=63 MCS) of ER membrane (Extended Data Fig. 7f). Thus, if equally distributed within MCS, the distance between bridges would be approximately 40 nm. However, the closest neighbour of each bridge was 15 nm away (Fig. 3c), indicating that ERMES bridges are not distributed homogenously, but rather in irregular clusters. The number of bridges found per MCS correlated with the ER membrane surface area in contact with mitochondria (Fig. 3g) and suggested that the maximum number of ERMES bridges is limited to approximately 12 bridges per 10,000 nm^2^ of ER surface area. Thus, while the distribution of bridges within the MCS appears to adopt no regular pattern and MCS morphologies are diverse, the number of ERMES bridges per surface area (Fig. 3g) and per MCS (Fig. 1c) appear to be constrained, indicative of spatial regulation of ERMES organization across MCS.

## Molecular model of ERMES

**Extended Data Figure 8:**
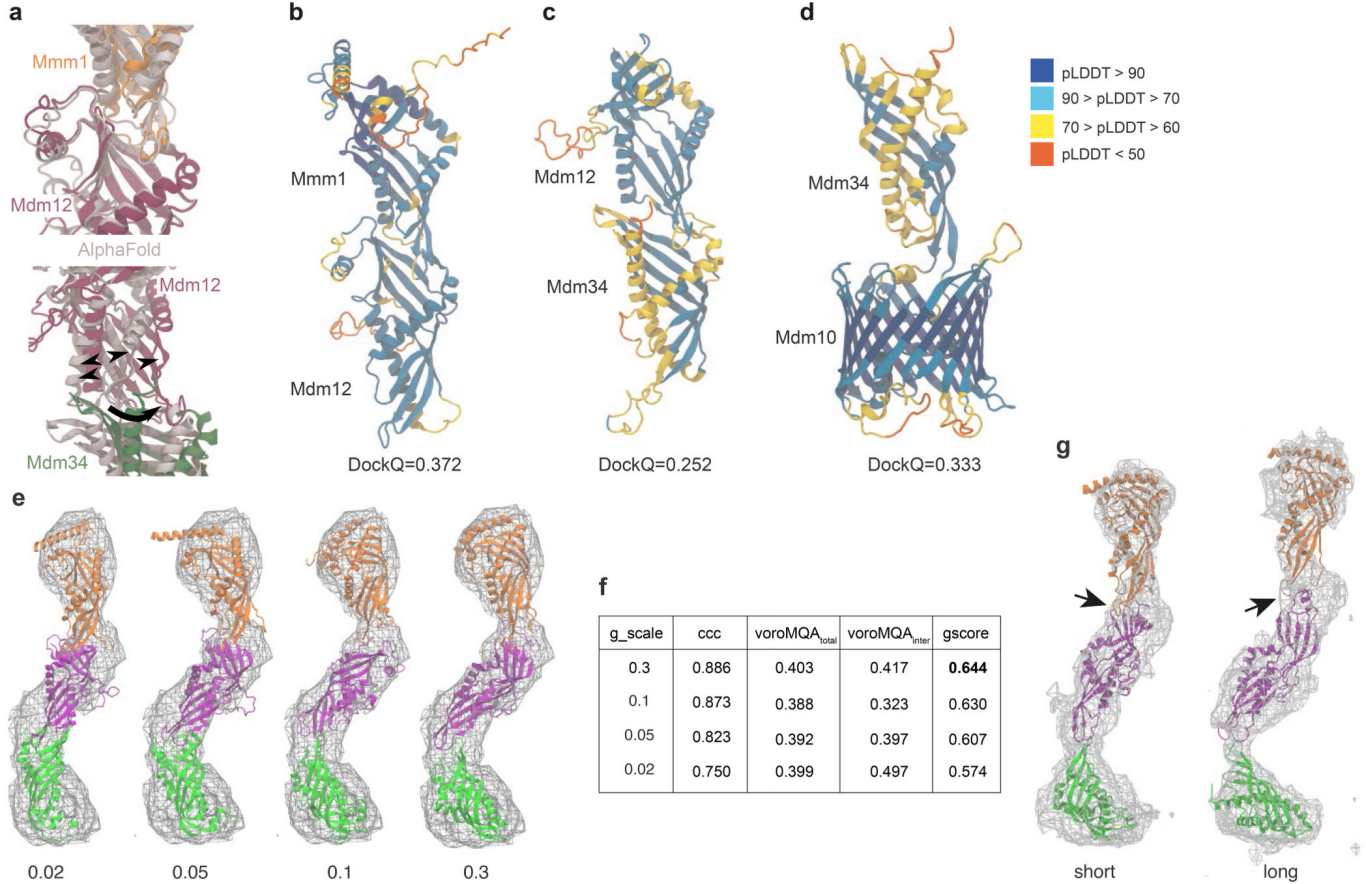
Integrative modelling of the ERMES complex. **a:** Differences between FD (in colour) and AF multimer (grey) predicted structures. While initial predictions of the complex structure using AF multimer^27,65^ and FD^26^ yield a nearly identical Mmm1-Mdm12 interface, the Mdm12-Mdm34 FD-interface shows a larger aperture (black arrowheads) which tends to close upon fitting to the cryo-ET STA map (black arrow). This difference between AF and FD highlights a lack of dynamic information as a current limitation of structural prediction. **b-d:** Quality estimation of the dimer predictions (Mmm1/Mdm12, Mdm12/Mdm34, Mdm34/Mdm10) using the FoldDock (FD) protocol. For each protein, the local pLDDT score is shown, together with the overall DockQ score of the dimer. For all protein-protein interfaces, the DockQ score is above the value generally considered to successfully predict heteromeric interfaces (DockQ>0.23)^26,65^. **e:** Final conformations of the trimeric Mmm1-Mdm12-Mdm34 complex, obtained from the MDFF simulations including 4 POPE lipids after fitting with different scaling factors (g_scale, numerical values given below models). The scaling factor determines the weight of the experimental STA map on the total molecular potential. **f:** Assessment of MDFF-derived models obtained using different scaling factors, shown in panel e. ccc refers to the cross-correlation coefficient between map and model, voroMQA_total_ and voroMQA^inter^ refer to the global voroMQA score and the component including only inter-subunit contacts, respectively. Based on the gscore which combines the assessment parameters (see Methods), g_scale=0.3 was considered best and is thus shown in other figures. **g:** Models obtained when the conformation of the Mmm1-Mdm12-Mdm34 complex was biased by MDFF into the short (left) and long (right) STA maps. The interaction between Mmm1 and Mdm12 (indicated by black arrows) appears to be diminished in the long conformation. In both cases, the model contained 4 lipids and the scaling factor was g_scale=0.1 See also Supplementary Table 1.

**Figure 4:**
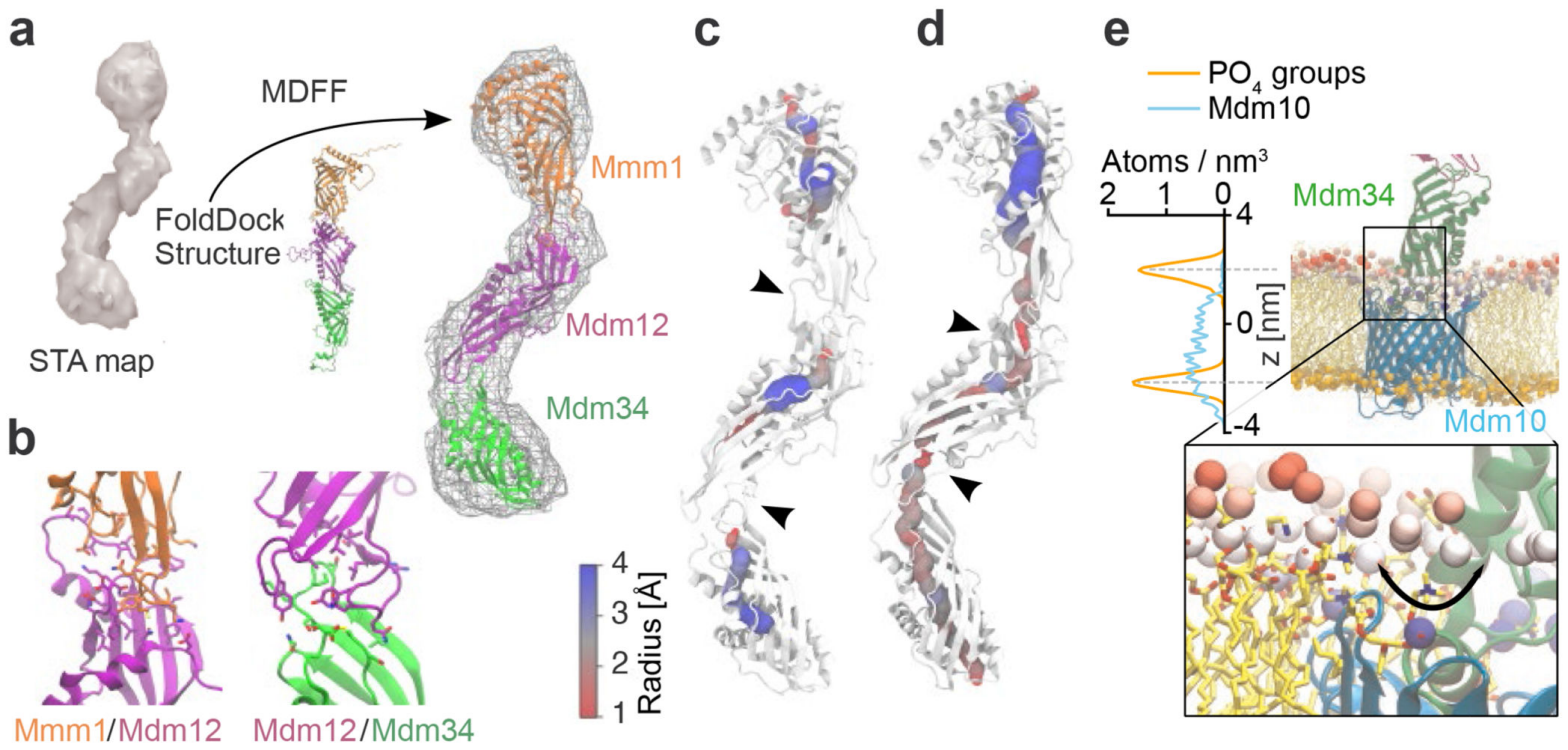
Integrative modelling of the ERMES complex. **a:** FD and MDFF approach^26,37^. The STA map was used to bias the conformation of the three SMP domains of ERMES. **b:** Predicted interfaces between Mmm1-Mdm12 (left), Mdm12-Mdm34 (right) in the model fit to the STA map. Interfacing residues (<3 Å) are shown as sticks. **c:** Average cavity of the heterotrimeric ERMES model with 4 POPE molecules. The three subunits show three distinct cavities which narrow at the subunit interfaces (black arrowheads). Cavity radii are indicated by sphere size and colour. **d:** Average cavity of the heterotrimeric ERMES model obtained with 12 POPE molecules. A continuous tunnel across subunit interfaces (black arrowheads) is observed as compared to c. The cavity is shown as an average of trajectory snapshots in which a full tunnel connects the two extremities of the complex (approximately 7% of total trajectory snapshots). Cavity radii are indicated by sphere size and colour. **e:** Hydrophobic mismatch of Mdm10 (cyan) embedded in an OMM-like bilayer. Phosphate groups of the upper leaflet are coloured according to their position along the z-axis (range: 10-25 Å from the bilayer centre, scale: blue to red). Left: Density profiles of the Mdm10 backbone (cyan) and membrane phosphate groups (orange), averaged over last 100 ns of the MDFF simulation. Inset: Close-up representation of the Mdm34-Mdm10 interface. Phosphate groups are represented as large Van der Waals spheres. Black arrow indicates nearness of Mdm34 cavity to phosphate groups

We next set out to investigate the molecular architecture of the ERMES complex. Our STA map suggests a zig-zag arrangement of three SMP domains (Fig. 2g). We sought to test if such an arrangement is compatible with the structural properties of the components. We predicted the structures of heterodimers (Mmm1-Mdm12 and Mdm12-Mdm34) using FoldDock (FD)^26^, an AlphaFold (AF)-based tool^27^, and assembled a heterotrimeric complex based on the sequential order of the components derived from our STA map and previous findings^16,20,21^ (Fig. 4a). FD predicted a sequential tail-to-head arrangement of the SMP domains of Mmm1, Mdm12 and Mdm34, with the head-terminal loop of one subunit interacting with the crevice formed by the next subunit (Extended Data Fig. 8a-c), similar to the interfaces observed in the X-ray structure of the Mmm1-Mdm12 dimer^16^.

Notably, rather than the zig-zag arrangement we observed *in situ*, the *in silico* FD prediction suggested a ‘linear’ conformation of the heterotrimeric complex, similar to the *in vitro* arrangement^16^ (Fig. 4a). To adjust the predicted complex structure to the experimental STA map which we had obtained from all bridges, we performed atomistic Molecular Dynamics Flexible Fitting (MDFF) (Fig. 4a, Extended Data Fig. 8e, f and Supplementary Video 8). Of note, even though the STA map does not provide secondary structure information, limiting the overall accuracy of our model, fitting to the STA map only moderately modified the intersubunit interfaces, which remained nearly in their initial FD-predicted conformations (Figs. 4a and b). Furthermore, our model does not completely occupy the STA map density around Mdm12 and Mdm34 (Fig. 4a). The extra density in the map remains unidentified but could correspond to the intrinsically disordered or dynamic protein regions that we excluded from modelling due to their poorly predicted structures. The density might also include the mNeonGreen protein with which Mdm34 was tagged, or auxiliary binding factors such as the regulatory proteins Gem1^21,28^, Tom7^20^, Mco6/Emr1^29^, or yet unknown factors.

**Extended Data Figure 9:**
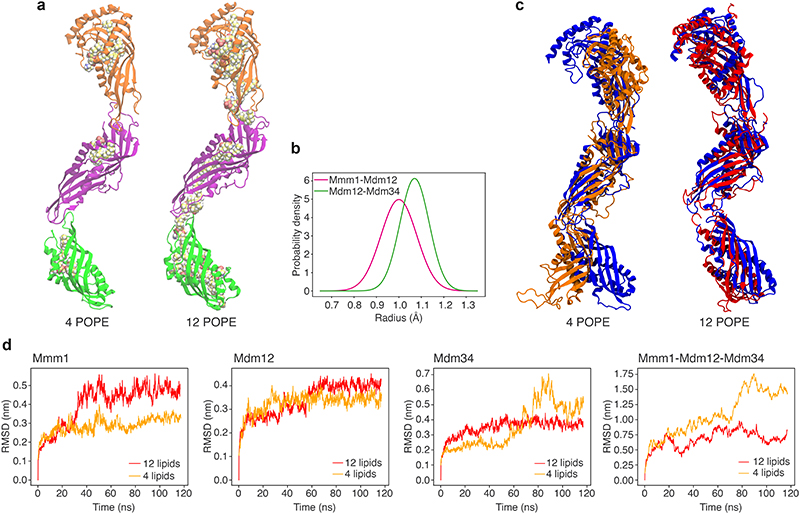
Effect of bound lipids on the ERMES complex model. **a:** Model of the Mmm1-Mdm12-Mdm34 complex obtained by MDFF when the starting complex contained 4 POPE lipid molecules (left) as compared to 12 POPE molecules (right). Note that the MDFF-derived model with 4 POPE molecules is also shown in Fig. 4a, b and d as well as in Extended Data Fig. 8e (g_scale=0.3), without visualisation of the lipids. **b:** Distributions of detected cavity radii at the interfaces between subunits Mmm1-Mdm12 (magenta) and Mdm12-Mdm34 (green). The radii were computed from frames taken over the last 100 ns of the MDFF simulation and are shown as probability density distributions. **c:** Structural comparison between the heterotrimeric complex obtained by MDFF with the STA map (blue), and the same complex after an additional unbiased MD simulation of 120 ns. The left model is with 4 lipids bound, the right model with 12 lipids bound. **d:** Root mean square deviations (RMSD) of individual subunits and of the heterotrimeric complex, measured over the additional 120 ns unbiased MD simulations of the MDFF-obtained heterotrimeric complex with either 12 or 4 lipids bound (red and orange, respectively). The end points (120 ns) of the red and orange plots correspond to red and orange conformations of the complex shown in d, respectively. The differences in the RMSD of Mmm1 with 12 vs. 4 lipids can be attributed to its N-terminal region, see also Supplementary Fig. 2.

In our final MDFF model, the conduit formed by the cavities of the SMP domains narrowed at the Mdm12-Mdm34 and Mmm1-Mdm12 interfaces (Fig. 4c). The model contained 4 lipid molecules based on the crystal structures of Mmm1 and Mdm12^16,25^ (Extended Data Fig. 9a). When we placed additional lipids in the cavities, the MDFF model suggested that a continuous lipid file may be possible through the interfaces (Fig. 4d, Extended Data Fig. 9a, b). These additional lipids could help to stabilize the zig-zag conformation of the complex (Extended Data Fig. 9c, d). Furthermore, the Mmm1-Mdm12 interface appeared diminished when the bridging distance was long (Extended Data Fig. 8g). These observations raise the possibility that while ERMES can form a continuous conduit between the membranes, the arrangement of the SMP domains could restrain the lipid pathway at the subunit interfaces, unlike in other tunnel-like lipid transfer proteins such as the VPS13 family, which form large unrestrained conduits^30^.

**Extended Data Figure 10:**
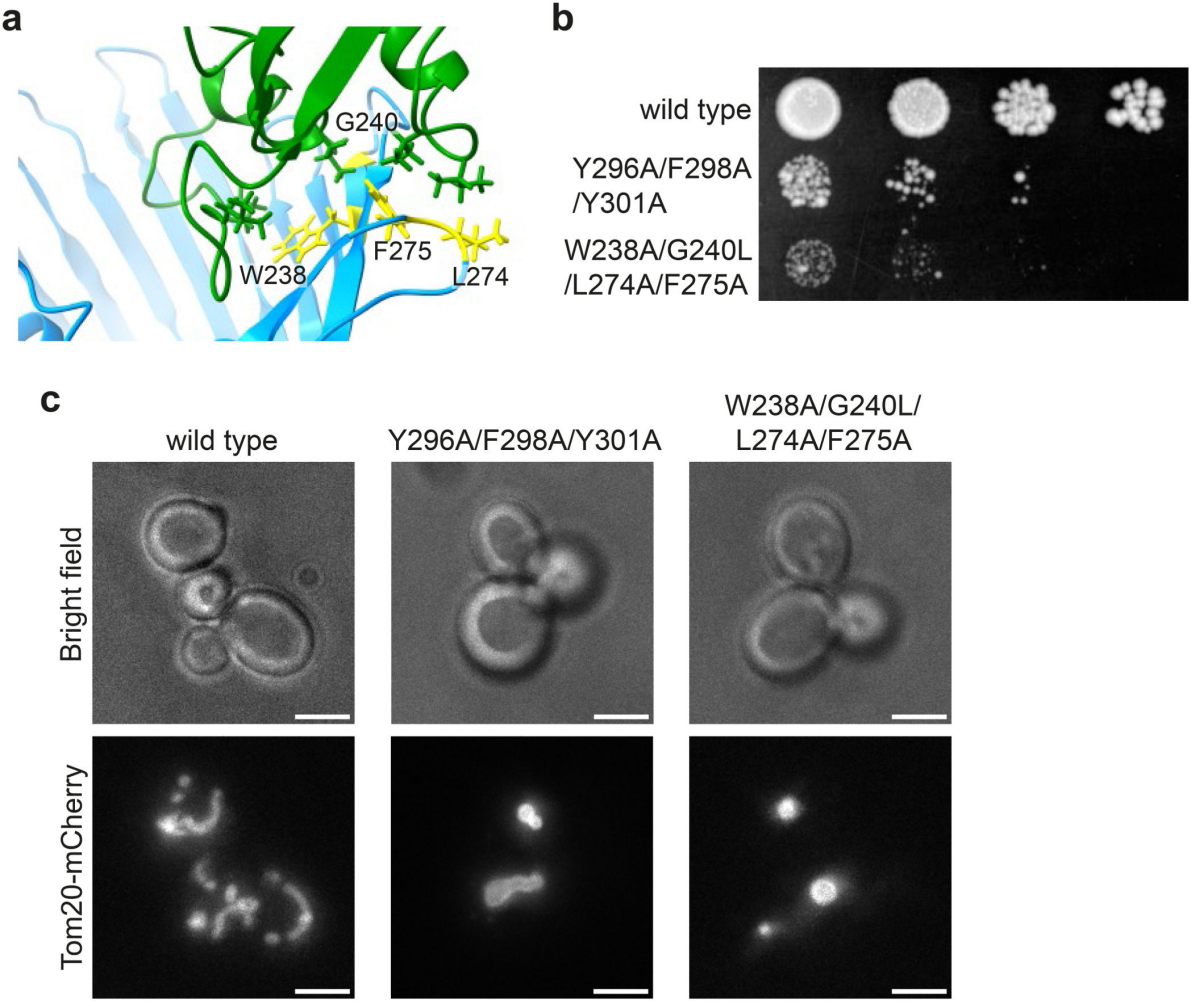
Mdm10 mutation in the predicted Mdm10-Mdm34 interface. **a:** MDFF-derived predicted interface between Mdm10 (blue) and Mdm34 (green). Residues in wild type Mdm10 that were mutated to Mdm10^W238A/G240L/L274A/F275A^ to disrupt the interface are indicated in yellow. The mutated residues are not involved in known interactions within the SAM complex^20,79^. **b:** Spot growth assay on YPD, comparing wild type, Mdm10^Y296A/F298A/Y301A^ (shown before to disrupt the interface between Mdm10 and Mdm34^20^) and Mdm10^W238A/G240L/L274A/F275A^. All three strains express Tom20-Cherry. **c:** Light microscopy of strains expressing wild type and mutant Mdm10. Tom20-mCherry is used as an indicator of mitochondrial morphology. Mdm10^Y296A/F298A/Y301A^ and Mdm10^W238A/G240L/L274A/F275A^ display a similar mitochondrial phenotype, indicative of ERMES complex disruption^4,20^. Scale bars are 3 μm. See also Supplementary Fig. 3.

We next built a full ERMES complex model which included the OMM protein Mdm10 using a similar protocol as for the heterotrimeric complex. Mdm10 was not part of our STA map, but it is known to interact with ERMES through Mdm34^20^. To test our full ERMES model, we mutated Mdm10 in its FD-predicted interface with Mdm34, which caused a phenotype similar to ERMES disruptions^4,20^ (Extended Data Fig. 10 and Supplementary Fig. 3). We then performed MDFF simulations of our full ERMES model with an explicit lipid bilayer mimicking the OMM. These simulations suggested that Mdm10 displays a hydrophobic mismatch on the cytosolic leaflet of the OMM, which deforms the bilayer around Mdm10 (Fig. 4e). Such lipid packing defects near the Mdm34 cavity (Fig. 4e, inset) might facilitate uptake or release of lipid molecules^31,32^.

## Discussion

In summary, combining quantitative FM, *in situ* structural biology and integrative modelling allowed us to seamlessly build a comprehensive and highly resolved model of ER-mitochondria MCS in yeast. In this model, ERMES distributes within MCS mostly as a discrete, two-membrane spanning complex of approximately 188 kDa in mass, with the three ERMES SMP domains arranged into a continuous structure between the two organelles. Whether the simultaneous stable association with both organelles is a general feature of lipid transfer complexes remains to be seen. The sequential arrangement of the SMP domains is likely to form a trajectory for lipids from one bilayer to the other, similarly to what has been suggested for large tunnel-like proteins such as VPS13^33^. The ERMES lipid conduit might be controllable at the interfaces between subunits. Based on our data, it is unlikely that ERMES shuttles between the membranes, as suggested for some other lipid transfer proteins^7,34,35^. We speculate that the structural variability of ERMES bridges in orientation, length and zig-zag conformation accommodates differences in intermembrane distance and could contribute to controlling lipid transfer. The exact mechanism regulating lipid passage through ERMES remains to be determined, but such a mechanism might facilitate specificity or directionality of lipid transport while being compatible with ERMES as an interorganelle tether.

## Methods

### Yeast cell culture and genetics

Liquid *Saccharomyces cerevisiae* (budding yeast) cultures were grown in synthetic complete medium without tryptophan with 2% glucose (SC-Trp) at 25°C for all experiments, except the growth assay. See supplementary information for a list of yeast strains used in this study. Genetic tagging with either EGFP, mNeonGreen or mCherry was done according to^38^, using plasmids pFA6a-EGFP-HIS3MX6, pFA6a-mCherry-kanMX4 and pFA6a-mNeonGreen-HIS3MX6^38,39^. Genomic mutants Mdm10^Y296A/F298A/Y301A^ and Mdm10^W238A/G240L/L274A/F275A^ were generated by CRISPR-Cas9 genome editing according to^40^. In brief, hybridized oligonucleotide pairs encoding guide RNA targeting sequences were ligated into the linearised sgRNA expression plasmid pML107^40^. Thereby generated plasmids pWK0353 and pWK0354 were amplified in *E. coli* Top10 cells. Haploid yeast strains expressing either Tom20-mCherry or both Tom20mCherry and Mdm10-GFP were co-transformed with purified plasmids and templates for homologous repair, which were PCR-amplified gBlocks (IDT) containing mutations of interest, following a published protocol^40^. See supplementary information for sequences of oligonucleotides and gBlocks. Genomic mutations were verified by sequencing of yeast colony PCR products. As suppressors arose quickly, mutants were reproduced by new transformation for every experiment. Mdm10^W238A/G240L/L274A/F275A^ was generated, sequence-verified and imaged three times, Mdm10^W238A/G240L/L274A/F275A^-EFGP was generated, sequence-verified and imaged once. Mdm10^Y296A/F298A/Y301A^ and Mdm10^Y296A/F298A/Y301A^-EGFP were each generated and sequence-verified once and imaged at least three times. To assay growth, liquid cultures of yeast strains were grown at 30°C to exponential phase. 5 μl of 10-fold serial dilutions were spotted on YPD plates and grown at 30°C for four days.

### Live fluorescence microscopy and quantification of the number of ERMES components per MCS

Quantification of the number of molecules of ERMES components per diffraction limited puncta was done as in^41^, according to the protocol described by^19^. Yeast cells expressing Cse4-EGFP Tom20-mCherry were mixed with cells expressing either Mmm1-EGFP, Mdm12-EGFP, Mdm34-EGFP or Nuf2-EGFP and then mounted on Concanavalin A coated 1.5 glass coverslips. The Tom20-mCherry signal was used to discriminate cells of the two strains. Stacked images (21 images over 4.2 μm z-height) were collected using the central 1024 x 1024 px of a QE80 Hamamatsu camera mounted on a Nikon Ti2 microscope equipped with a Nikon Plan Apo VC 100x/1.40 NA oil objective, controlled by the NIS-Elements software. A NIJI LED light source with 470 nm and 550 nm LEDs was used for excitation with the filter sets 49002-ET-EGFP (FITC/Cy2) with ET470/40x, T495lpxr and ET525/50m for green fluorescence and 49005-ET-DSRed (TRITX/Cy3) with ET545/30x, T570lp and ET620/60m for red fluorescence. Stacked images were collected of green fluorescence before red fluorescence imaging. For each experimental repeat, images of Cse4-EGFP, Mmm1-EGFP, Mdm12-EGFP, Mdm34 -EGFP and Nuf2 were collected on the same day. Z-stacked images of individual cells (either expressing one of the ERMES components or Cse4-EGFP Tom20-mCherry cells in anaphase-telophase) were cropped manually using Fiji^42^ and then subjected to the spotquant analysis pipeline, which includes background subtraction, identification and masking of fluorescent spots prior to measurement of fluorescence intensity^19^. The Python3 package spotquant was accessed at https://github.com/apicco/spotquant. As Cse4-EGFP Tom20-mCherry expressing cells were mixed with each of the other four strains, cropped images of Cse4-EGFP labelled kinetochores from the four mixes were merged and used for further analysis. To determine the median number of molecules per punctum of ERMES components, the median fluorescence intensity of Cse4-EGFP from that day was assumed to correspond to 80 EGFP molecules and was used to transform the median fluorescence intensity of Mmm1-EGFP, Mdm12-EGFP and Mdm34-EGFP into numbers of molecules. To validate the workflow, the ratio of Nuf2-EGFP molecules with respect to Cse4-EGFP molecules was measured and found to be 3.5:1, in close agreement with the published ratio of 3.6:1^36,43^. Live microscopy images shown in Fig. 1a and b are maximum projection images through z-stacks. Images shown in Extended Data Fig. 1 are single planes from z-stacks. For estimating the correlation between fluorescence intensities of Mdm34-EGFP with Mmm1-mCherry and Mdm34-EGFP with Mdm12-mCherry, the respective strains were mounted on Concanavalin A coated no. 1 glass coverslips. Stacked images (17 images over 3.4 μm z-height) were collected using the central 1024 x 1024 px of a Photometrics Prime BSI sCMOS camera mounted on a Nikon Eclipse Ti2 microscope equipped with an CFI Apochromat TIRF 100x/1.49 NA oil objective, controlled by the NIS-Elements software. A Lumencor SpectraX light source (Chroma) with 470 nm and 555 nm LEDs was used for excitation, with quad band filter set 89000 ET Sedat Quad (Chroma) including ET490/20x, 89100bs, ET525/36m for green fluorescence and ET555/25x, 89100bs, ET605/52m for red fluorescence as well as an emission filter wheel (Nikon) set to 535 nm for green fluorescence and 638 nm for red fluorescence. Z-stacked images of individual cells were cropped manually using Fiji^42^. We modified spotquant to compare the fluorescence intensities on GFP and mCherry colocalising patches. The modified software, the code used to generate the data, and the fluorescence intensity values in Extended Data Fig. 1 can be downloaded here: https://github.com/apicco/spotquant/tree/b10.

### Imaging and quantification of the total mitochondrial fluorescence per cell

Live fluorescence microscopy of yeast cells expressing Tom20-mCherry and variants of Mdm10, or Tom20-mCherry and variants of Mdm10-EGFP, was done with the same microscopy setup as used for the correlation of fluorescence intensities of ERMES components described above. The corresponding live microscopy images shown in Extended Data Fig. 10 and Supplementary Fig. 3 are maximum projection images through z-stacks. Total mitochondrial fluorescence per cell was quantified using an ImageJ/FIJI macro (totalFluorPerCell, https://github.com/mwozn/totalFluorPerCell) which processed z-stack images of yeast cells expressing Tom20-mCherry and either Mdm10-EGFP or Mdm10^W238A/G240L/L274A/F275A^-EGFP. See Supplementary Figure S3b for the results of the analysis. The macro prompts the user to define individual cells as round ROI. Then the macro applies an adaptive threshold (https://sites.google.com/site/qingzongtseng/adaptivethreshold) to the image stack’s red fluorescence channel, ROIs are cropped, and cleared outside of the ROI; producing a binarized image of mitochondria highlighted by Tom20-mCherry. Next, the pixel intensity of the green fluorescence channel is measured within pixels corresponding to mitochondria in the binarized image for each z-slice. For background subtraction, the round, user-defined ROI subtracted by the pixels corresponding to the mitochondrial selection was considered as the cell background fluorescence per each z-slice. A corrected total mitochondrial fluorescence value was calculated from the measured integrated density, subtracted by the product of the measured area (pixels) and the mean intensity of the cell background per z-slice. The corrected total mitochondrial fluorescence value of each z-slice was summed to obtain total mitochondrial fluorescence per cell.

### Cryo-FIB milling of yeast cells, cryo-CLEM and cryo-ET

Log-phase cultures of yeast expressing Mdm34-mNeonGreen and Dnm1-mCherry were pelleted and resuspended in SC-Trp with 15% dextran (w/v, Sigma 40 kDa Mw) before applying to glow discharged Quantifoil R2/2 Cu 200 mesh grids, manually back blotting and plunging into liquid ethane using a manual plunger with temperature control^44^. Lamellae of vitrified yeast cells were prepared as described by^17,45^ using a Scios DualBeam FIB/SEM microscope (FEI) equipped with a Quorum PP3010T cryo-FIB/SEM preparation system. To improve lamellae stability, micro-expansion joints were made^46^. Cryo-FM imaging of lamellae was done on a Zeiss Axio Imager.M2m microscope equipped with a Linkam CMS196 cryo-stage and an Axiocam 503 mono CCD camera (Zeiss). Bright field images of the grids were collected using an EC Plan-Neofluar 10x/0.30 (Zeiss) objective lens. High-magnification fluorescence images of lamellae were collected with a LD EC Epiplan-Neofluar 100x/0.75 DIC M27 (Zeiss) objective lens. A Zeiss Colibri 7 LED light source with 475 nm and 555 nm LEDs was used for excitation with the filter sets 38 HE eGFP (Zeiss) for green fluorescence (Mdm34-mNeonGreen) and 63 HE mRFP (Zeiss) for red fluorescence (Dnm1-mCherry). Room humidity was kept below approx. 25% during cryo-FM. Lamellae which contained Mdm34-mNeonGreen signals were used in the next steps of cryo-ET tilt series acquisition. A fraction of tilt series was collected from lamellae regions which contained Dnm1-mCherry signal in addition to Mdm34-mNeonGreen (see ‘Segmentation and Ultrastructural Analysis’). Cryo-ET tilt series were collected on a Titan Krios microscope (Thermo Fisher) operated at 300 kV using a BioQuantum energy filter (slit width 20 eV) and a K3 direct electron detector (Gatan). SerialEM^47^ was used for acquiring montaged maps of the lamellae and for tilt series acquisition. Low magnification maps (39.4 Å/px) were correlated with cryo-FM images by 2D rigid fitting using the Icy plug-in ecCLEM^48^. As landmarks for registering cryo-FM and cryo-EM images, features of the lamella and yeast cell outlines, as well as holes and imperfections in the surrounding carbon support film which were visible in both cryo-FM and cryo-EM images were used. In this way regions containing Mdm34-mNeonGreen puncta were identified. In a second step, the resulting composite images were correlated to medium magnification anchor maps (10.7 Å/px), using features recognisable at low and intermediate magnification. The medium magnification maps were used to navigate tilt series acquisition. Tilt series images were acquired at 1° increment over a ±56° or in a few cases a ±60° range in groups of 4 using a dose-symmetric tilt scheme^49,50^ in superresolution mode at a pixel size of 1.342 Å and a defocus range from -3.5 to -6.0 μm. Total exposure time was adjusted to maintain a total dose per tilt image of 1.3 e^-^/Å^2^ using a dose rate of approximately 25 e^-^/px/s. Exposures were fractionated as four frames acquired as LZW-compressed TIFF images without normalisation and without binning. See also Extended Data Table 1.

### Tomogram Reconstruction and Subtomogram Averaging

Frames were gain corrected, aligned, dose-weighted and binned to a pixel size of 2.684 Å with the preprocessing script from the subTOM package^51^ which executes IMOD’s alignframes and ctfplotter functions^52^. Tilt series were aligned using IMOD’s etomo with patch tracking and 2D CTF correction applied by phase-flipping in IMOD. Tomograms were reconstructed at bin 2 (resulting in 5.368 Å pixel size) using etomo by either weighted back-projection for subtomogram averaging or simultaneous iterative reconstruction technique (SIRT) for tomograms used for manual coordinate picking of the bridges’ ER and OMM anchor points, segmentations and for figures. For improved visibility in some figure panels, 3D median filtering was applied to tomograms and in some cases to virtual tomographic slices. Bridge structures were manually picked using Dynamo^53^ as dipole models of ER and OMM anchor points. Dipole coordinates from Dynamo were converted to a MOTL file for use in subTOM using MATLAB (MathWorks) code https://github.com/mwozn/DYNAMO_dipoles_to_MOTL. The central point and axis between ER and OMM anchor points were used to define the coordinates for subtomogram extraction using the subTOM package which utilizes MATLAB functions adapted from TOM^54^, AV3^55,56^, and Dynamo^53^. The presented STA of all bridges consists of 1098 subtomograms, extracted from 51 tomograms acquired at regions of lamella corresponding to ERMES puncta imaged by cryo-FM. See Extended Data Fig. 3 for a schematic of the STA procedure. For initial reference generation, a random spin rotation was applied to each subtomogram to minimise the effect of the preferential missing wedge upon subtomogram alignment. Subtomogram alignment was inspected between alignment iterations using the UCSF Chimera^57^ plug-in Place Object^58^. The initial number of manually picked subtomograms in Dynamo was 1133. Subtomograms which aligned poorly and instead ‘migrated’ to align to the ER or OMM membranes, were found by visually inspecting the position of the STA map placed back at the positions of the subtomograms within each tomogram. There were 25 such misaligned subtomograms and they were removed from the list of subtomogram positions used for STA before the subTOM analysis was re-run. In addition, 10 subtomograms which belonged to ER-peroxisome MCS were removed at this point, thus the final data set consisted of 1098 subtomograms. The resolution of the resulting STA map was estimated to be 29 Å from the Fourier Shell Correlation (FSC) curve at 0.143, generated according to Chen *et al.*^59^, implemented through subTOM^51^ (Extended Data Fig. 4a). For FSC analysis, the STA half maps were masked to avoid the membranes, which were also excluded during alignment, to influence the assessment (Extended Data Fig. 4b). For display of the STA map in Fig. 2 and Supplementary Video 1, the map was rendered in UCSF ChimeraX^60^.

### Subtomogram averaging of short and long bridges

To obtain STA maps of the short and long halves of the data set of bridge structures, the data set was split into two equal groups, each containing 549 subtomograms, separated based on the length of the dipole models picked initially in Dynamo^53^ (https://github.com/mwozn/getDipoleLength_binByLength). Each group was then subjected to the same alignment procedure as described above and in Extended Data Fig. 3. The final STA maps obtained from this alignment procedure were aligned to the STA map of the complete dataset using subTOM for easier comparison. For display of the STA maps in Extended Data Fig. 5, the maps were rendered in UCSF ChimeraX^60^.

### Analysis of the angles between bridge and OMM, and between bridge and ER membrane

STA was used to align the subtomograms composing the bridge STA map to the OMM or the ER. The zenith rotational angle was compared between the original alignment and the alignment to the OMM or the ER, to obtain the angle between the z-axis of the bridge and the normal of the OMM or the ER. For this alignment, the subtomograms aligned before (Extended Data Fig. 3) were repositioned so that the OMM- or ER-proximal end of the bridge was centred at the pivot point of the STA alignment search. The subtomograms were masked to include the OMM and a part of the bridge (Extended Data Fig. 6c), or to include the ER and a part of the bridge (Extended Data Fig. 6h). The alignment search consisted of 5° zenith tilt-steps, from 0 to +/-60°, without in-plane rotation. The differences in zenith rotation of each subtomogram before and after this alignment were calculated in MATLAB and analysed in R. For display of the resulting STA maps in Extended Data Fig. 6, the maps were rendered in UCSF ChimeraX^60^.

### Nearest neighbour analysis

For the nearest neighbour analysis, the Euclidean distances between all the centres of subtomograms were calculated with MATLAB (https://github.com/mwozn/MOTL_neighbourAnalysis), and for each subtomogram the minimal distance was identified as the one to the nearest neighbour. Three bridges from two tomograms were excluded from this analysis because these tomograms contained only one or two bridges; therefore, 1095 bridges from 49 tomograms were compared by nearest neighbour analysis. For determining the distance to the nearest neighbour of the ER and OMM anchor points, subtomograms were first re-centred on the ER or OMM anchor points, respectively, and the Euclidean distances to the nearest neighbours were determined as for the centres. Nearest neighbour analysis was also performed after classifying bridges into short and long categories; for this, the coordinates of the separately aligned short and long bridges were recombined using MATLAB and the TOM^54^ software toolbox, then the Euclidean distances between the centres of aligned short and long bridge subtomograms were analysed within their respectively shared tomogram space. In this way, for each bridge we determined whether its nearest neighbour was a bridge from the short or long category.

### Numerical data analysis

Data was analysed using MATLAB, GraphPad Prism and R (R Core Team, 2021). For figure panels, the data was plotted using ggplot2^61^ and raincloud plots^62^ within R, and GraphPad Prism.

### Segmentation and ultrastructure analysis

Segmentation models of mitochondrial membranes, ER and peroxisomes were drawn manually in IMOD^52^ and rendered for figure and video preparation in UCSF ChimeraX^60^. To determine the surface area of the contact site, the ER membrane in proximity to the OMM was segmented in IMOD and its surface area was measured using imodinfo^52^. The UCSF Chimera^57^ plug-in Place Object^58^ was used to place back the STA maps into tomographic coordinates and determine the number of ERMES bridges per surface area, as well as the number of bridges belonging to the short and long categories per MCS. Contact site surface area and number of ERMES bridges per surface area were determined in 49 of the 51 ER-mitochondria MCS containing tomograms. Two tomograms were excluded from this analysis because segmentation of the ER membrane was not possible due to poor quality and visibility. Some tomograms contained multiple separate patches of ER membrane connected the mitochondrion; these were counted as separate MCS, resulting in a total of N=63 MCS. The analysis in Extended Data Fig. 7d did not require segmentation and was done on N=64 MCS. Of the 51 tomograms containing ER-mitochondria MCS which were used for subtomogram averaging, 16 were acquired in regions that in addition to Mdm34-EGFP also contained Dnm1-mCherry. We did not notice any difference in appearance, ultrastructure, number of bridges per MCS, or surface area, between tomograms from regions of Mdm34-mNeonGreen only, and tomograms from regions with both Mdm34-mNeonGreen and Dnm1-mCherry.

### Structural modelling

For structure prediction, the sequences were modified as follows. *Mmm1*: Truncated the ER-interacting N-terminal side after the helical segment (residues: 1-161), as in the crystal structure^16^. *Mdm12*: Based on the structure from^25^, the used sequence comprises only part of the loop between residues 73 and 115, adding 5 residues to each side of the broken loop (73-78) and (110-115). The loop has been shown to not be necessary for function^25^. *Mdm34*: Only residues 1-200 were used, corresponding to the SMP domain. The C-terminal part is expected to be partially disordered based on secondary structure prediction and its interaction with the rest of the complex is currently unknown. *Mdm10*: Three regions facing the intermembrane space and one facing the cytoplasmic side of the membrane are likely disordered or partially disordered and were not included in the used sequence (residues 95-128, 219-224, 323-406, 453-473). None of the removed loop regions is expected to strongly affect the overall architecture of the model or is interacting with other modelled subunits^25,63^. The structure of the predicted models (Extended Data Fig. 8) shows a good or even excellent quality for the majority of the structures (pLDDT > 70), with the exception of some parts of Mdm34.

The ERMES complex structure was predicted by combining dimer predictions using the FoldDock (FD) protocol^26^, specifically Mmm1/Mdm12 and Mdm12/Mdm3 for the heterotrimeric complex, and additionally Mdm34/Mdm10 for the full tetrameric complex (Extended Data Fig. 8a-d). We combined the subunits by aligning the common proteins of the predictions. To account for the different conformations adopted by Mdm34 in the Mdm12/Mdm34 and Mdm34/Mdm10 dimers, for the full tetrameric complex we performed a Targeted Molecular Dynamics simulation and changed the conformation of part of the subunit to match both interfaces. The simulation was performed using Mdm34 from the Mdm10/Mdm34 predicted structure, using implicit Generalized Born solvent (ɛ=80), added secondary structure restraints, and maintaining the regions interfacing with Mdm10 restrained to their initial positions with a harmonic force constant of 2 kcal mol^-1^ Å^-2^ (residues 1-31, 75-130, 147-152). The simulation was performed using Charmm36m force-field parameters^64^ Langevin dynamics at *T* = 310 K and a time step of 1 fs. The combination of the predictions from FD by aligning the central subunits (e.g. Mmm1/Mdm12-Mdm12/Mdm34) results in a roughly linear arrangement of subunits. In addition to FD, we performed structural prediction using Alphafold-Multimer^27,65^. The dimeric structures are predicted in the same head-to-tail conformation, yielding an overall similar architecture. The FD structure shows a larger aperture at the Mdm12/Mdm34 interface (Extended Data Fig. 8a).

Since the FD-predicted structure is devoid of lipids, we generated two different lipid-bound states. In the first model, a total of 4 POPE lipids were included in the Mmm1/Mdm12/Mdm34 complex (2:1:1) using lipid molecules from the resolved crystal structures (for Mmm1 and Mdm12)^16,25^ or added by similarity with other subunits in Mdm34. In the second model, 8 additional POPE lipids were manually docked in the internal cavities observed in the heterotrimeric complex, for a total of 12 lipids (4 for each subunit).

### Molecular dynamics flexible fitting

We next biased the structures obtained with FD according to the STA maps using a Molecular Dynamics Flexible Fitting approach (MDFF)^37^. To do so, all systems were solvated in water (TIP3P model) and sodium and chloride ions were added to give an ionic strength of approximately 120 mM. In addition, the full tetrameric complex with 4 POPE lipids in the SMP domains, was embedded in a previously equilibrated POPC:POPE:POPI (50:35:15) membrane resembling an Outer Mitochondrial Membrane (OMM)-like composition^66^ using Charmm-GUI^67^. The model systems corresponding to the heterotrimeric assembly and the full complex comprised approximately 50,000 and 330,000 atoms, respectively. All the simulations were performed using Charmm36m force-field parameters, Langevin dynamics at *T* = 310 K, P=1 atm and a time step of 1 fs. Long-range electrostatics were treated by means of the Particle Mesh Ewald (PME) method^68^. Secondary structure (helices and b-sheet dihedral psi/chi) and chirality restraints were added to avoid that the introduced forces would cause large distortions of the protein structures.

For the heterotrimeric complex, the solvated model systems (containing either 4 or 12 POPE molecules) were initially thermalized and equilibrated while keeping the protein backbone atoms restrained to their original positions. For the full tetrameric complex, this protocol was further extended (30 ns) to allow for full equilibration of the membrane now in the presence of the Mdm10 subunit. Then, in all cases, the structures were biased via MDFF simulations that were run until convergence. The length of the performed simulations is reported in Supplementary Table 1. Convergence was assessed in all cases by following the behaviour of the RMSD (root mean square deviation of the protein atomic positions from the initial structure) over the time of the simulations. RMSD plots obtained from the simulations with optimal g_scale values (see below) are shown in Supplementary Fig. 1. The experimental STA maps were used to generate grid potentials with voxel sizes of 5.368 Å derived from the STA maps (from all-bridges as well as short and long classes). These densities were trimmed and centred to contain only the region of interest. Additionally, we partially reduced the noise of the STA maps by applying a gaussian filter. In all MDFF simulations, the grid-based potentials were applied to Mmm1, Mdm12, and the solvent-exposed region of Mdm34.

A crucial parameter in these simulations is the scaling factor (g_scale). It plays a key role in the flexible fitting approach since it determines how strongly the protein model will be fitted to the STA map; i.e., it controls the weight of the experimental STA map on the total molecular potential. Since it has been reported that large g_scale values can induce overfitting by artificially increasing the cross-correlation between the atomic models and the STA density maps^69,70^, we investigated the impact of using various scaling factors (g_scale values of 0.3, 0.1, 0.05, and 0.02 kcal mol^-1^, Extended Data Fig. 8e,f) on the structures sampled by the MDFF simulations. For every simulation, the optimal g_scale value was inferred using a normalized global score (*gscore*) that combines information from two equally-weighted metrics: *gscore* = 1/2 (*ccc+voroMQA*). The first term in this equation, *ccc*, is the widely used cross-correlation coefficient and it quantifies the goodness-of-fit between the model-derived and experimental densities (Supplementary Fig. 1). The second one, *voroMQA*, globally estimates (i) the integrity and atomic quality (clashes, atomic deviations from expected positions, etc.) of the individual proteins that form the oligomeric complex and (ii) the quality of the quaternary structure of the assembly by assessing the complementarity of the residues at the interface between the different subunits. The latter term was calculated using the VoroMQA package^71^. According to our definition, a higher *gscore* value will be indicative of a higher-quality complex structure. Hence, for each individual fitting procedure, we selected the protein model corresponding to the g_scale parameter resulting in the highest *gscore* (Extended Data Fig. 8f).

All the simulations were performed using NAMD Git2021-11-23 with CUDA acceleration^72^. Analyses and system preparation were performed using VMD^73^ and ChimeraX^64^. The detection of cavitites and tunnels was performed using CAVER 3.0^74^ with default values for all parameters. All cavities/tunnels that were identified were clustered using the hierarchical average-link algorithm in order to remove redundant information. The remaining identified tunnels present a bottleneck equal or larger to the probe radius of 0.9 Å. In each system analyzed, the search for tunnels was performed on a conformational ensemble containing 1000 equispaced snapshots (covering 100 ns of dynamics) that were collected from the last part of the corresponding trajectories. The images shown in Fig. 4c and d correspond to the longest tunnels found along the three subunits within the conformational ensemble, the cavity radius at each coordinate is shown as an average of the trajectory snapshots.

### Statistics and Reproducibility

All stable yeast strains used in this study and listed in supplementary information were live imaged at least three times from independently grown liquid cultures. Yeast cells expressing Mdm10 mutants were imaged every time they were freshly generated (see ‘Yeast cell culture and genetics’). The cryo-ET data was acquired on yeast cells plunge-frozen from at least three independently grown liquid cultures. 51 tomograms acquired of Mdm34-mNeonGreen puncta contained ER-mitochondrial MCS (examples shown in Fig. 2b and Extended Data Fig. 2a-f) and 9 contained ER-peroxisome MCS (examples shown in Extended Data Fig. 2g, h).

## Extended Data

**Extended Data Table 1:** Cryo-ET data collection and processing. Parameters of cryo-ET data set collection used for subtomogram averaging, resulting in the STA map of bridge structures.

	ERMES bridges
	EMD-16873
**Cryo-ET data collection and** **processing**	
Microscope	Titan Krios
Magnification	33000 ×
Voltage (kV)	300
Detector	Gatan K3 in superresolution mode
Energy filter	yes
Electron exposure per tilt series	approx. 140-150
(e^-^/Å^2^)	
Defocus range (μm)	-3.5 to -6.0
Pixel size (Å)	1.342
Tilt range (min, max, increment)	-56°, +56°, 1°
Tilt scheme	Dose-symmetric
Frame number	4
Tomograms used for STA (no.)	51
Initial subtomograms (no.)	1133
Final subtomograms (no.)	1098
Map resolution (Å) at FSC threshold 0.143	29
Map resolution range (Å)	-

## Supplementary Material

Extended Data

Supplementary Information

## Data Availability

Data generated in this study have been deposited at the Electron Microscopy Data Bank^75^ (http://www.ebi.ac.uk/emdb) under accessions EMD-15355, EMD-16871, EMD-16872 and EMD-16873. Raw cryo-ET data has been deposited at EMPIAR^76^ (https://www.ebi.ac.uk/empiar) under accession EMPIAR-11462. Live fluorescence microscopy data and integrative molecular models have been deposited on http://www.zenodo.org/ with DOIs 10.5281/zenodo.6784812, 10.5281/zenodo.7392153, 10.5281/zenodo.7753491 and 10.5281/zenodo.7736245. Source data are provided with this paper. The SpotQuant package^19^ used for quantification of fluorescence intensities is available at https://github.com/apicco/spotquant and https://github.com/apicco/spotquant/tree/b10. The subtomogram averaging package subTOM^51^ is available at https://github.com/DustinMorado/subTOM. Other code available is for convertion of Dynamo dipole model coordinates to a MOTL file https://github.com/mwozn/DYNAMO_dipoles_to_MOTL, calculation and classification of bridge lengths from dipole ER- and OMM-anchor points https://github.com/mwozn/getDipoleLength_binByLength, nearest-neighbour analysis https://github.com/mwozn/MOTL_neighbourAnalysis, and fluorescent protein levels on mitochondria https://github.com/mwozn/totalFluorPerCell.
