# Supplementary material for: In situ architecture of the ER-mitochondria encounter structure
Source: Nature. Author manuscript; Available in PMC 2023 Jun 1. (PMC7614606; doi:10.1038/s41586-023-06050-3)
Supplement: Extended Data [file EMS173552-supplement-Extended_Data.pdf]

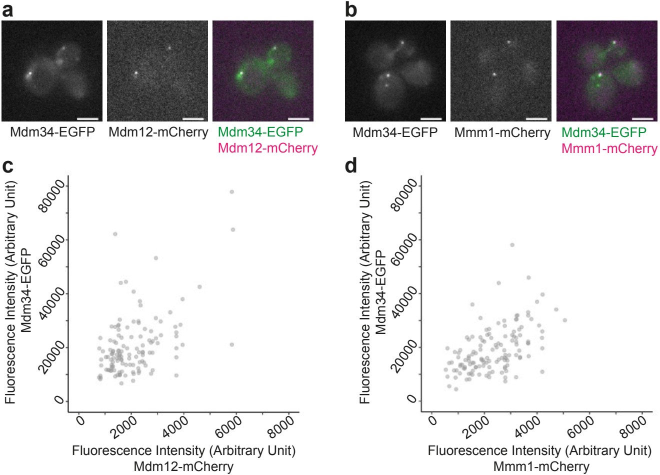
a
Mdm34-EGFP
Mdm12-mCherry
Mdm34-EGFP
Mdm12-mCherry
b
Mdm34-EGFP
Mmm1-mCherry
Mdm34-EGFP
Mmm1-mCherry
c
Fluorescence Intensity (Arbitrary Unit)
Mdm34-EGFP
0
20000
40000
60000
80000
0
2000
4000
6000
8000
Fluorescence Intensity (Arbitrary Unit)
Mdm12-mCherry
d
Fluorescence Intensity (Arbitrary Unit)
Mdm34-EGFP
0
20000
40000
60000
80000
0
2000
4000
6000
8000
Fluorescence Intensity (Arbitrary Unit)
Mmm1-mCherry

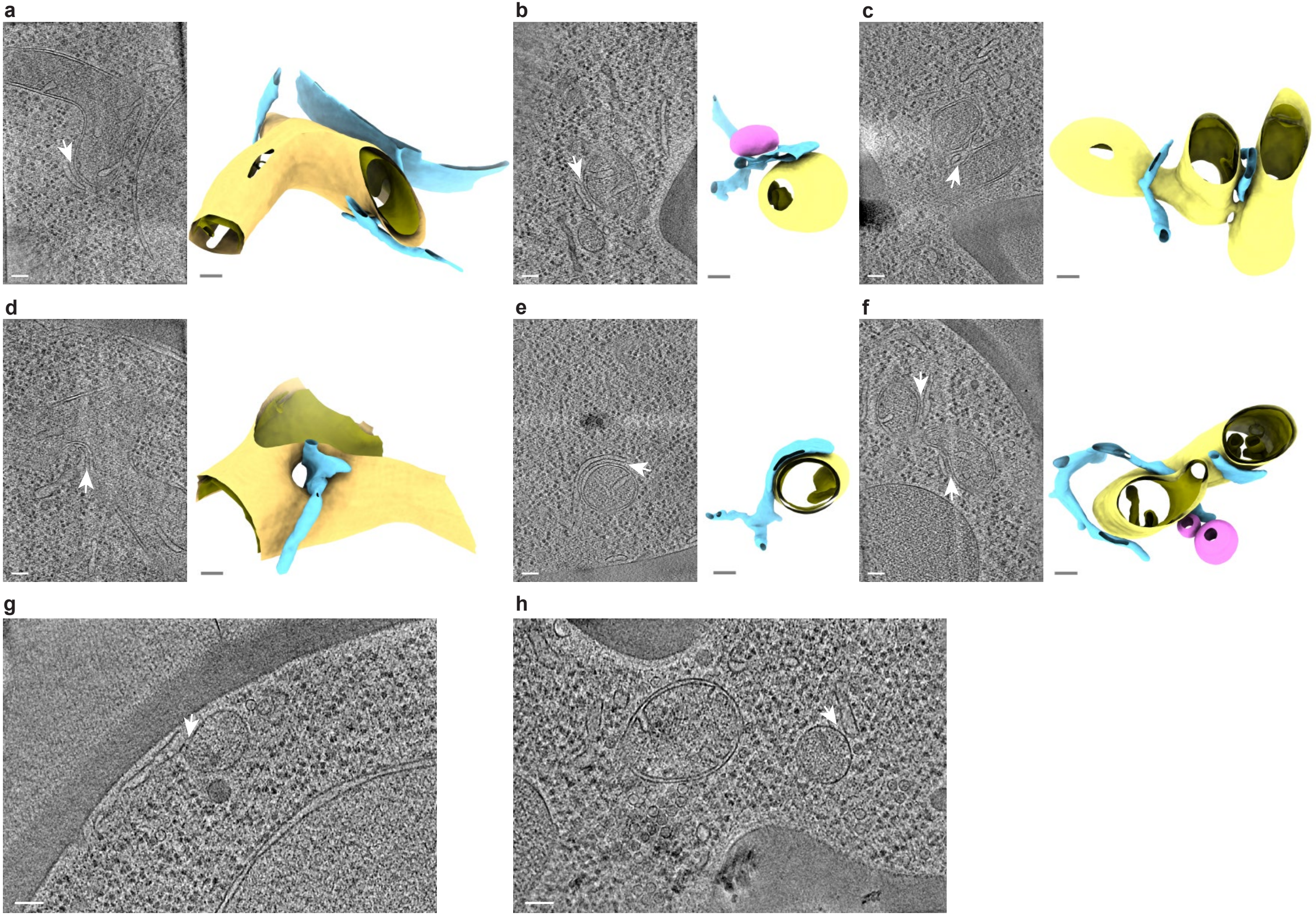

a
b
c
d
e
f
g
h

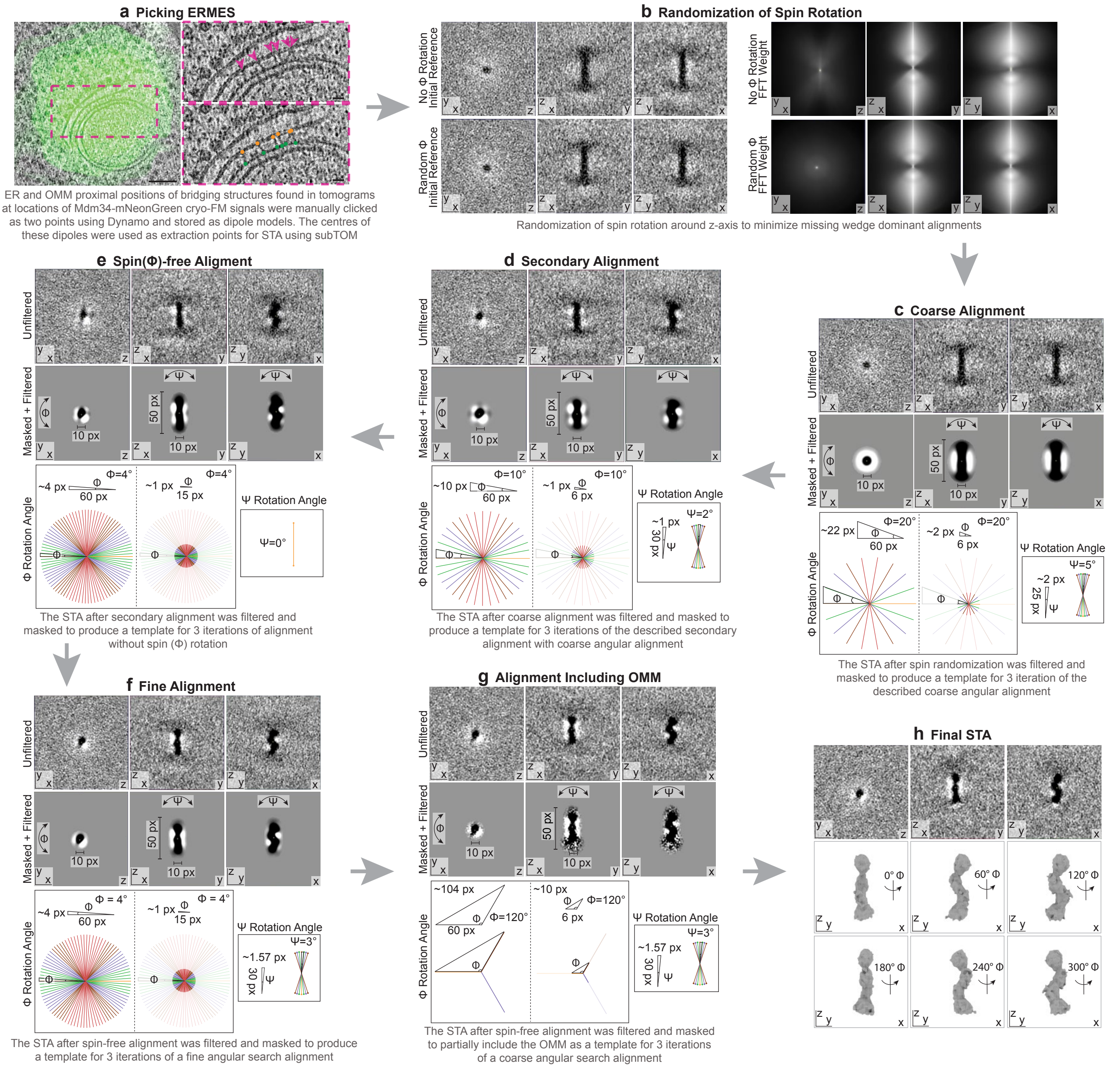
a Picking ERMES
ER and OMM proximal positions of bridging structures found in tomograms at locations of Mdm34-mNeonGreen cryo-FM signals were manually clicked as two points using Dynamo and stored as dipole models. The centres of these dipoles were used as extraction points for STA using subTOM
b Randomization of Spin Rotation
No Φ Rotation Initial Reference
Random Φ Initial Reference
No Φ Rotation FFT Weight
Random Φ FFT Weight
Randomization of spin rotation around z-axis to minimize missing wedge dominant alignments
c Coarse Alignment
Unfiltered
Masked + Filtered
10 px
50 px
Φ Rotation Angle
~22 px
60 px
Φ=20°
~2 px
6 px
Ψ Rotation Angle
Ψ=5°
~2 px
25 px
The STA after spin randomization was filtered and masked to produce a template for 3 iteration of the described coarse angular alignment
d Secondary Alignment
~10 px
60 px
Φ=10°
~1 px
6 px
Ψ=2°
~1 px
30 px
The STA after coarse alignment was filtered and masked to produce a template for 3 iterations of the described secondary alignment with coarse angular alignment
e Spin(Φ)-free Alignment
~4 px
60 px
Φ=4°
~1 px
15 px
Ψ=0°
The STA after secondary alignment was filtered and masked to produce a template for 3 iterations of alignment without spin (Φ) rotation
f Fine Alignment
Φ = 4°
~1.57 px
30 px
Ψ=3°
The STA after spin-free alignment was filtered and masked to produce a template for 3 iterations of a fine angular search alignment
g Alignment Including OMM
~104 px
60 px
Φ=120°
~10 px
6 px
The STA after spin-free alignment was filtered and masked to partially include the OMM as a template for 3 iterations of a coarse angular search alignment
h Final STA
0° Φ
60° Φ
120° Φ
180° Φ
240° Φ
300° Φ

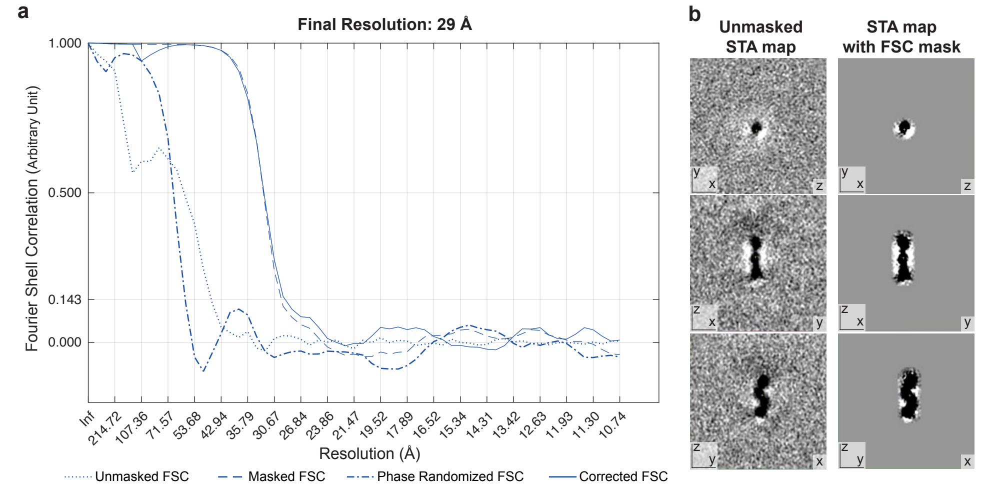

a
Final Resolution: 29 Å
1.000
0.500
0.143
0.000
Fourier Shell Correlation (Arbitrary Unit)
Inf
214.72
107.36
71.57
53.68
42.94
35.79
30.67
26.84
23.86
21.47
19.52
17.89
16.52
15.34
14.31
13.42
12.63
11.93
11.30
10.74
Resolution (Å)
Unmasked FSC
Masked FSC
Phase Randomized FSC
Corrected FSC
b
Unmasked STA map
STA map with FSC mask
y
x
z
z
x
y
z
y
x

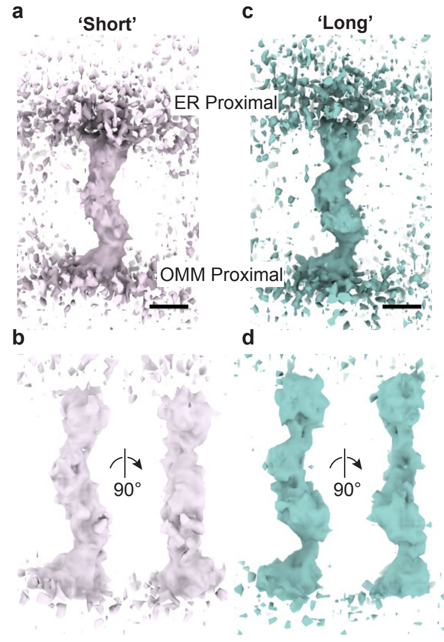

a
'Short'
c
'Long'
ER Proximal
OMM Proximal
b
d
90°
90°

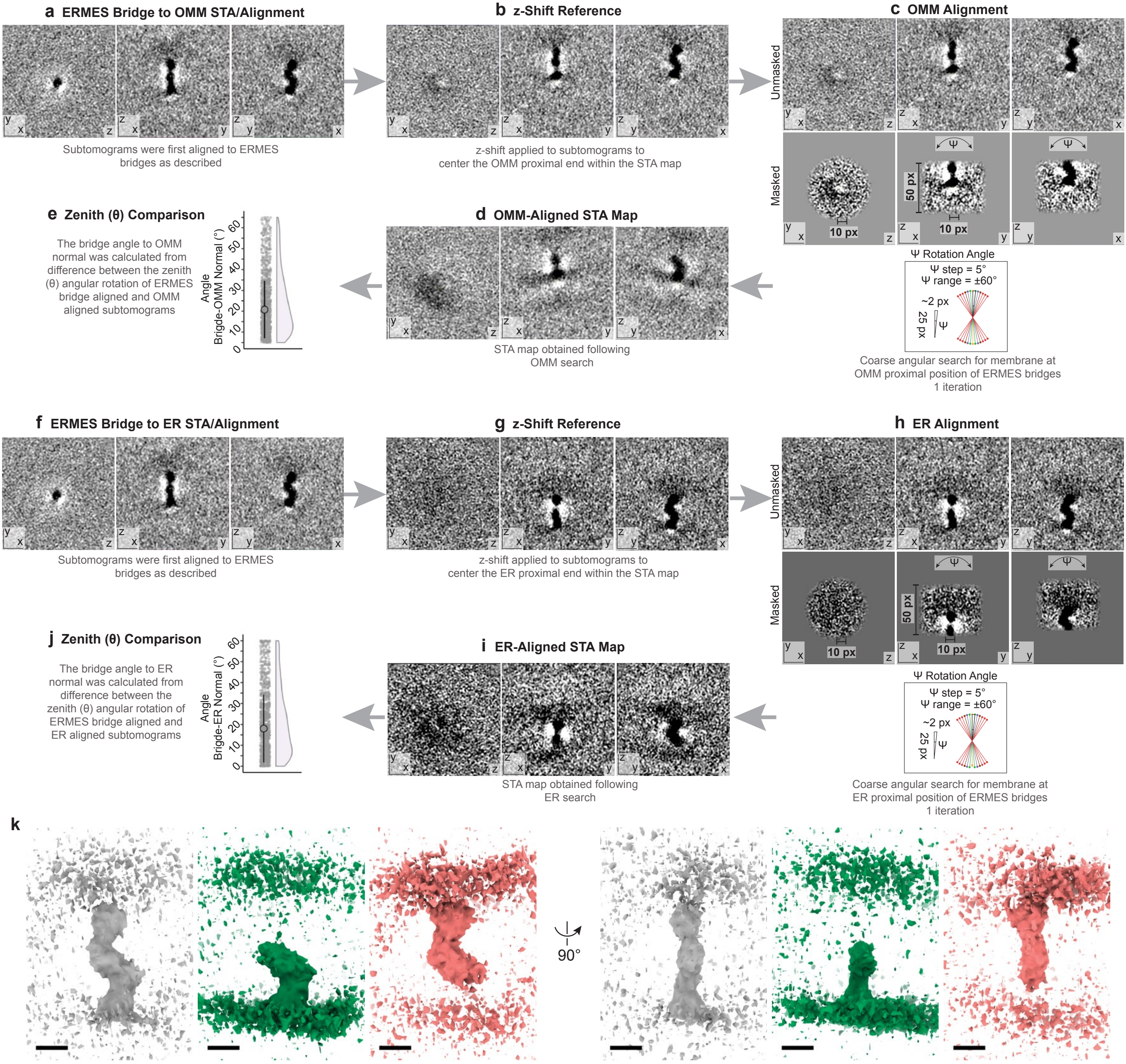

a ERMES Bridge to OMM STA/Alignment
Subtomograms were first aligned to ERMES bridges as described
b z-Shift Reference
z-shift applied to subtomograms to center the OMM proximal end within the STA map
c OMM Alignment
Unmasked
Masked
50 px
10 px
Ψ Rotation Angle
Ψ step = 5°
Ψ range = ±60°
~2 px
25 px
Coarse angular search for membrane at OMM proximal position of ERMES bridges
1 iteration
d OMM-Aligned STA Map
STA map obtained following OMM search
e Zenith (θ) Comparison
The bridge angle to OMM normal was calculated from difference between the zenith (θ) angular rotation of ERMES bridge aligned and OMM aligned subtomograms
Angle Brigde-OMM Normal (°)
f ERMES Bridge to ER STA/Alignment
Subtomograms were first aligned to ERMES bridges as described
g z-Shift Reference
z-shift applied to subtomograms to center the ER proximal end within the STA map
h ER Alignment
Unmasked
Masked
50 px
10 px
Ψ Rotation Angle
Ψ step = 5°
Ψ range = ±60°
~2 px
25 px
Coarse angular search for membrane at ER proximal position of ERMES bridges
1 iteration
i ER-Aligned STA Map
STA map obtained following ER search
j Zenith (θ) Comparison
The bridge angle to ER normal was calculated from difference between the zenith (θ) angular rotation of ERMES bridge aligned and ER aligned subtomograms
Angle Brigde-ER Normal (°)
k
90°

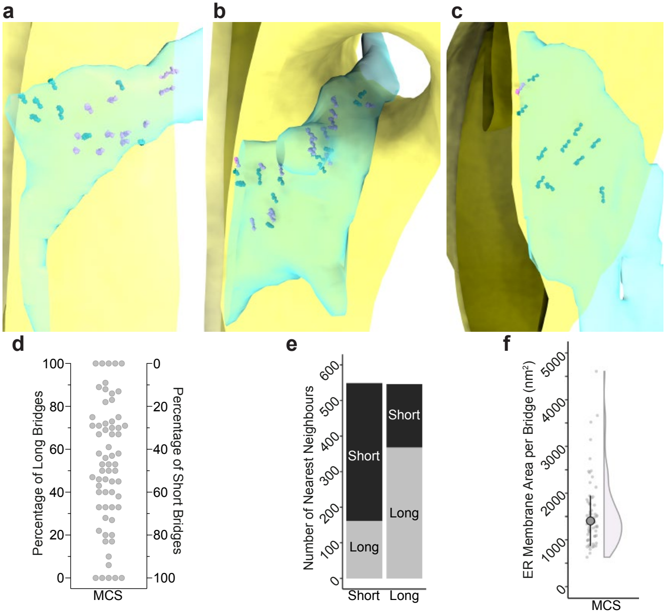
a
b
c
d
Percentage of Long Bridges
Percentage of Short Bridges
100
80
60
40
20
0
MCS
e
Number of Nearest Neighbours
0
100
200
300
400
500
600
Short
Long
Short
Long
Short
Long
f
ER Membrane Area per Bridge (nm^2)
0
1000
2000
3000
4000
5000
MCS

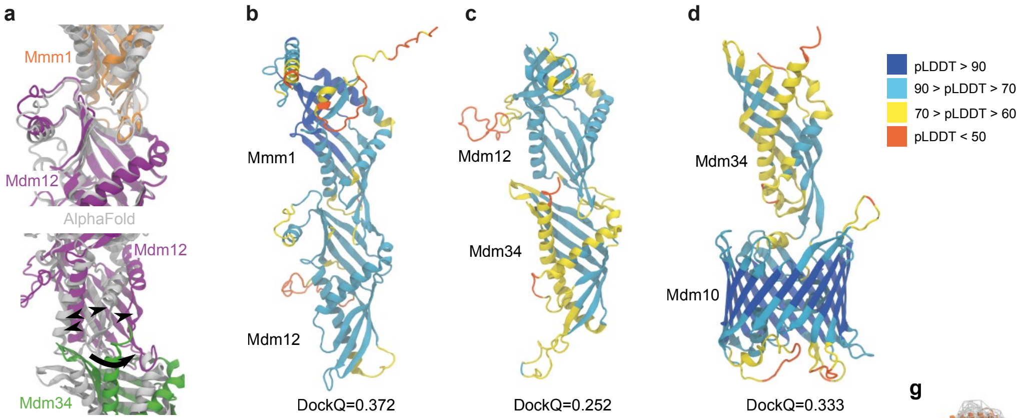

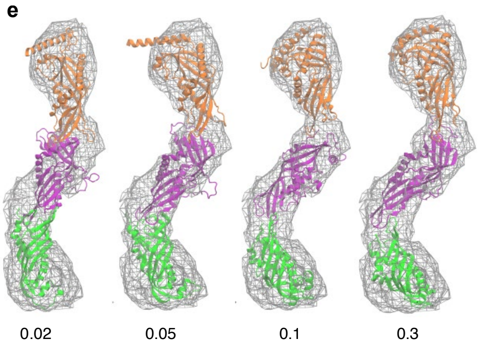

f

g_scale	ccc	voroMQA$_{total}$	voroMQA$_{inter}$	gscore
0.3	0.886	0.403	0.417	**0.644**
0.1	0.873	0.388	0.323	0.630
0.05	0.823	0.392	0.397	0.607
0.02	0.750	0.399	0.497	0.574

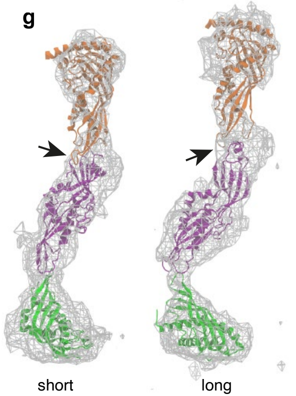

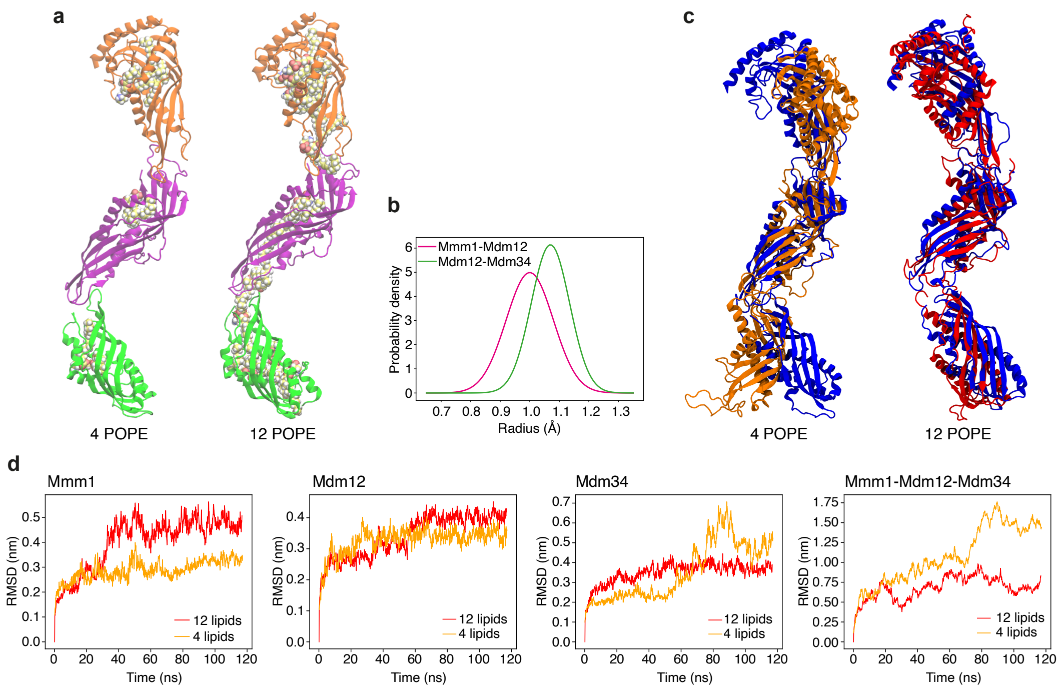

a
4 POPE
12 POPE
b
Mmm1-Mdm12
Mdm12-Mdm34
Probability density
Radius (Å)
c
4 POPE
12 POPE
d
Mmm1
Mdm12
Mdm34
Mmm1-Mdm12-Mdm34
RMSD (nm)
Time (ns)
12 lipids
4 lipids

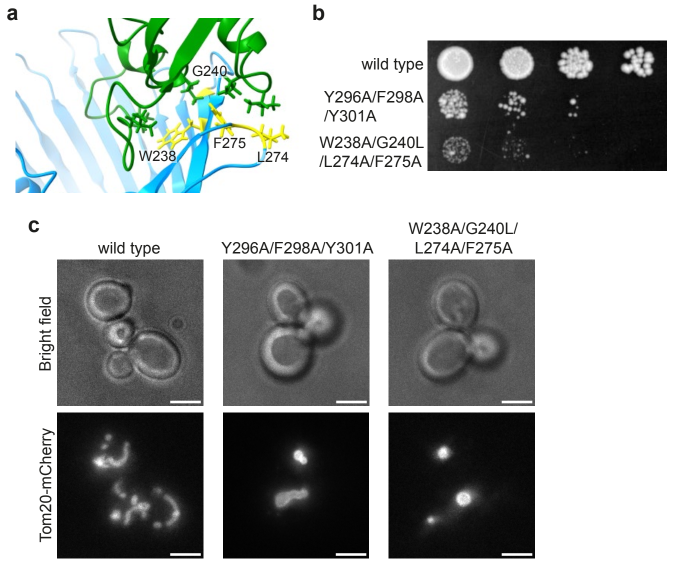
a
G240
W238
F275
L274
b
wild type
Y296A/F298A
/Y301A
W238A/G240L
/L274A/F275A
c
wild type
Y296A/F298A/Y301A
W238A/G240L/
L274A/F275A
Bright field
Tom20-mCherry

	**ERMES bridges**<br>EMD-16873
**Cryo-ET data collection and processing**	
Microscope	Titan Krios
Magnification	33000 x
Voltage (kV)	300
Detector	Gatan K3 in superresolution mode
Energy filter	yes
Electron exposure per tilt series ($e^{-}/Å^2$)	approx. 140 - 150
Defocus range (μm)	-3.5 to -6.0
Pixel size (Å)	1.342
Tilt range (min, max, increment)	-56°, +56°, 1°
Tilt scheme	Dose-symmetric
Frame number	4
Tomograms used for STA (no.)	51
Initial subtomograms (no.)	1133
Final subtomograms (no.)	1098
Map resolution (Å)<br>at FSC threshold 0.143	29
Map resolution range (Å)	-
