# Supplementary material for: In situ architecture of the ER-mitochondria encounter structure
Source: Nature. Author manuscript; Available in PMC 2023 Jun 1. (PMC7614606; doi:10.1038/s41586-023-06050-3)
Supplement: Supplementary Information [file EMS173552-supplement-Supplementary_Information.pdf]

Supplementary information

In situ architecture of the ER-mitochondria encounter structure

Authors:

Michael R. Wozny[1,7], Andrea Di Luca[2], Dustin R. Morado[1,3,8], Andrea Picco[4], Rasha Khaddaj[5], Pablo Campomanes[2], Lazar Ivanović[5,6], Patrick C. Hoffmann[1,9], Elizabeth A. Miller[1], Stefano Vanni[2]*, Wanda Kukulski[1,5]*

*Correspondence to:

stefano.vanni@unifr.ch

wanda.kukulski@unibe.ch

Affiliations:

[1]MRC Laboratory of Molecular Biology, Francis Crick Avenue, Cambridge CB2 0QH, UK

[2]Department of Biology, University of Fribourg, 1700 Fribourg, Switzerland

[3]SciLifeLab, Tomtebodavägen 23, 171 65 Solna, Sweden

[4]Department of Biochemistry, University of Geneva, Quai Ernest Ansermet 30, 1211 Geneva, Switzerland

[5]Institute of Biochemistry and Molecular Medicine, University of Bern, Bühlstrasse 28, 3012 Bern, Switzerland

[6]Graduate School for Cellular and Biomedical Sciences, University of Bern, Mittelstrasse 43, 3012 Bern, Switzerland

Present addresses:

[7]Department of Anatomy and Cell Biology, McGill University, Montreal, H3A 0C7, Canada

[8]Max Planck Institute of Biochemistry, 82152 Martinsried, Germany

[9]Max Planck Institute of Biophysics, 60438 Frankfurt am Main, Germany

<u>Supplementary materials and methods:</u>

Yeast strains used for live cell fluorescence microscopy

Mdm34-mNeonGreen, Tom20-mCherry (WKY0401): MATa, his3Δ200, leu2-3,112, ura3-52, lys2-801, MDM34-mNEONGREEN::HIS3MX6, TOM20-mCHERRY::kanMX4

Nuf2-EGFP (WKY0337): MATα, his3Δ200, leu2-3,112, ura3-52, lys2-801, NUF2-EGFP::HIS3MX6

Cse4-EGFP, Tom20-mCherry (WKY0421): MATα, his3Δ200, leu2-3,112, ura3-52, lys2-801, CSE4-EGFP::HIS3MX6, TOM20-mCHERRY::kanMX4

Mdm12-EGFP (WKY0119): MATα, his3Δ200, leu2-3,112, ura3-52, lys2-801, MDM12-EGFP:: HIS3MX6

Mdm34-EGFP (WKY0432): MATα, his3Δ200, leu2-3,112, ura3-52, lys2-801, MDM34-EGFP:: HIS3MX6

Mmm1-EGFP (WKY0433): MATα, his3Δ200, leu2-3,112, ura3-52, lys2-801, MMM1-EGFP:: HIS3MX6

Mdm34-EGFP, Mdm12-mCherry (WKY0605): MATα, his3Δ200, leu2-3,112, ura3-52, lys2-801, MDM34-EGFP::HIS3MX6, MDM12-mCHERRY::kanMX4

Mdm34-EGFP, Mmm1-mCherry (WKY0606): MATa, his3Δ200, leu2-3,112, ura3-52, lys2-801, MDM34-EGFP::HIS3MX6, MMM1-mCHERRY::kanMX4

Tom20-mCherry (WKY0310): MATα, his3Δ200, leu2-3,112, ura3-52, lys2-801, TOM20-mCHERRY::kanMX4

Mdm10-EGFP, Tom20-mCherry (WKY0609): MATα, his3Δ200, leu2-3,112, ura3-52, lys2-801, MDM10-EGFP::HIS3MX6, TOM20-mCHERRY::kanMX4

Yeast strain used for cryo-CLEM /cryo-ET

Mdm34-mNeonGreen, Dnm1-mCherry (WKY0400): MATa, his3Δ200, leu2-3,112, ura3-52, lys2-801, MDM34-mNEONGREEN::HIS3MX6, DNM1-mCHERRY::kanMX4

Oligonucleotides used for guide RNA plasmid cloning to target MDM10

See Methods for procedure.

Oligonucleotide pair to generate plasmid pWK0353:
GATCTTAAAGTTGTCGAACAACCGGTTTTAGAGCTAG
CTAGCTCTAAAACCGGTTGTTCGACAACTTTAA

Oligonucleotide pair to generate plasmid pWK0354:
GATCTGTCTTGGCCGAATATGTGGGTTTTAGAGCTAG
CTAGCTCTAAAACCCACATATTCGGCCAAGACA

Sequences of gBlocks co-transformed with the guide plasmids

To generate Mdm10$^{W238A/G240L/L274A/F275A}$ and Mdm10$^{W238A/G240L/L274A/F275A}$-EGFP, yeast strains WKY0310 and WKY0609, respectively, were co-transformed with plasmid pWK0353 and the following gBlock:
TTACACAATTTCCTTACCACGCCTTCCAAGTTTAACACCTCACTGTACAATAATTC
TTCGTTGTCGCTTGGTGCTGAATTTGCTCTTCTGCTAGTATCACTTTCCCCCGGAT
GTTCAACAACACTGAGGTATTATACGCATAGCACCAACACTGGTCGTCCATTGAC
TCTAACCCTTTCCTGGAACCCAGCGGCCGGCCATATATCCTCCACATATTCGGCC
AAGACAGGGACAAATTCTACTTTTTGCGCGAAGTATGATTTTAATCTTTATTCGA
TTGAATCAAATCTTTCATTTGGGTGCGAATTTTGGCAAAAA

In one experiment, two gBlocks were sequentially used to generate Mdm10$^{W238A/G240L/L274A/F275A}$. First, Mdm10$^{W238A/G240L}$ was generated by co-transforming yeast strain WKY310 with plasmid pWK0353 and the following gBlock:
TTACACAATTTCCTTACCACGCCTTCCAAGTTTAACACCTCACTGTACAATAATTC
TTCGTTGTCGCTTGGTGCTGAATTTGCCCTTCTACTGGTATCACTGTCTCCGGGTT
GCAGTACGACGCTGCGTTATTACACACATTCTACAAACACAGGACGACCACTAA
CTTTGACATTATCTTGGAATCCATTATTCGGCCATATATCCTCCACATATTCGGCC
AAGACAGGGACAAATTCTACTTTTTGCGCGAAGTATGATTTTAATCTTTATTCGA
TTGAATCAAATCTTTCATTTGGGTGCGAATTTTGGCAAAAA

The resulting cells were then co-transformed with plasmid pWK0354 and the following gBlock to generate Mdm10$^{W238A/G240L/L274A/F275A}$:

GGGTTAGTAAGTTTAAGCCCCGGTTGTTCGACAACTTTAAGATATTACACACATT
CTACAAACACAGGACGACCACTAACTTTGACATTATCTTGGAATCCAGCTGCCGG
GCATATCTCTAGTACCTATTCTGCGAAGACGGGGACAAATTCTACTTTTTGCGCG
AAGTATGATTTTAATCTTTATTCGATTGAATCAAATCTTTCATTTGGGTGCGAATT
TTGGCAAAAAAAGCATCATTTGCTTGAAACCAATAAAAACAATAATGATAAATT
AGAACCAATCTCCGACGAATTGGTTGATATAAATCCAAACAGCAGAGCGACTAA
AC

To generate Mdm10$^{Y296A/F298A/Y301A}$ and Mdm10$^{Y296A/F298A/Y301A}$-EGFP, yeast strains WKY0310 and WKY0609, respectively, were co-transformed with plasmid pWK0354 and the following gBlock:

GGGTTAGTAAGTTTAAGCCCCGGTTGTTCGACAACTTTAAGATATTACACACATT
CTACAAACACAGGACGACCACTAACTTTGACATTATCTTGGAATCCATTATTCGG
CCATATATCCTCAACCTATAGTGCTAAAACAGGAACTAACTCAACTTTCTGCGCA
AAGGCGGATGCGAATTTAGCCTCGATTGAATCAAATCTTTCATTTGGGTGCGAAT
TTTGGCAAAAAAAGCATCATTTGCTTGAAACCAATAAAAACAATAATGATAAAT
TAGAACCAATCTCCGACGAATTGGTTGATATAAATCCAAACAGCAGAGCGACTA
AAC

Supplementary Figure 1:

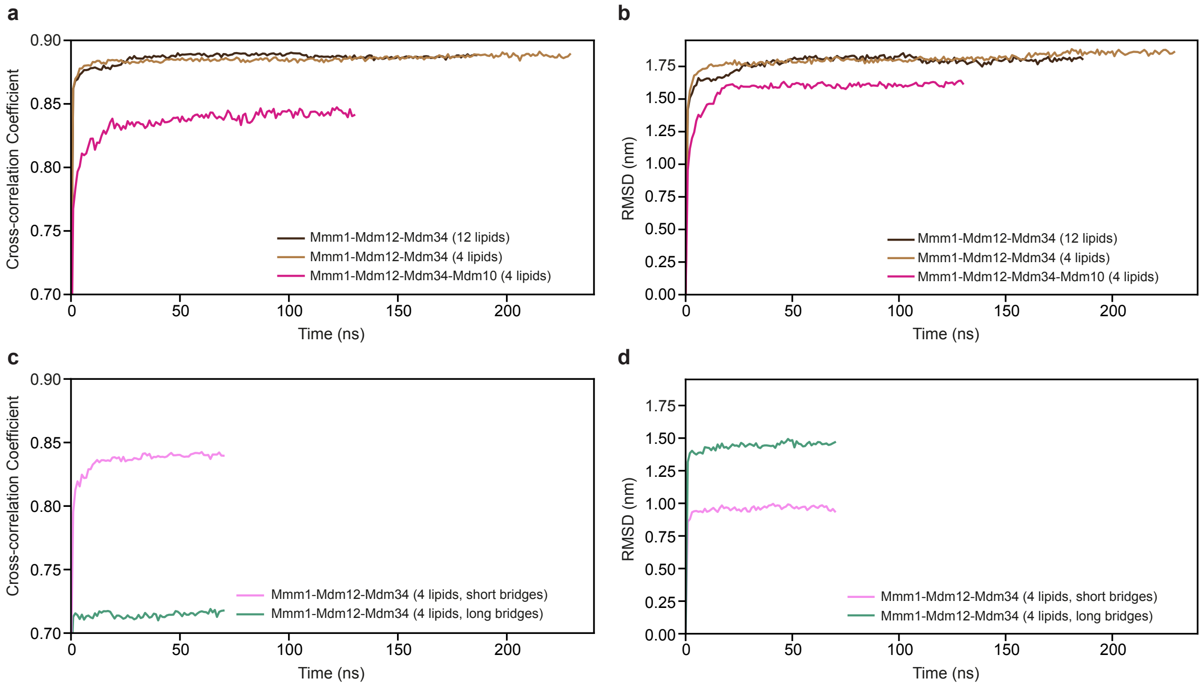

Supplementary Figure 1 legend: Cross-correlation coefficients (a, c) calculated from comparing simulated (filtered to 30 Å resolution) and experimental STA maps, and root mean square deviations (RMSD) (b, d) of the respective models, plotted over the time of the respective MDFF experiment. **a:** Cross-correlation between the STA map of all bridges and the heterotrimeric complex with 12 lipids (dark brown), the heterotrimeric complex with 4 lipids (light brown), and the heterotetrameric complex with 4 lipids (magenta), respectively. **b:** RMSD of the heterotrimeric complex with 12 lipids (dark brown), the heterotrimeric complex with 4 lipids (light brown), and the heterotetrameric complex with 4 lipids (magenta), respectively, during MDFF into the STA of all bridges. **c**: Cross-correlation between the STA map of the short bridges (Extended Data Fig. 5a) and the heterotrimeric complex with 4 lipids (pink) and the STA map of the long bridges (Extended Data Fig. 5b) with the heterotrimeric complex with 4 lipids (teal). **d:** RMSD of the heterotrimeric complex with 4 lipids during MDFF into the short (pink) and long (teal) STA map.

Supplementary Figure 2:

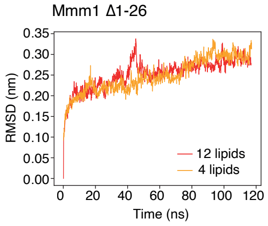

Supplementary Figure 2 legend: Related to Extended Data Fig. 9d. Root mean square deviations (RMSD) of Mmm1 when not considering the helix formed by the first 26 residues of our model. In the full-length Mmm1 sequence, these 26 residues are preceded by the N-terminal transmembrane domain of Mmm1 which anchors ERMES to the ER membrane. We have not modelled the N-terminal transmembrane domain of Mmm1 due to lack of structural information, likely leading to significant movement of this helix in unbiased MD simulations. The RMSD was measured over the additional 120 ns unbiased MD simulations of the MDFF-obtained heterotrimeric complex with either 12 or 4 lipids bound (red and orange, respectively). The end points (120 ns) of the red and orange plots correspond to red and orange conformations of the complex, respectively, shown in Extended Data Fig 9c.

Supplementary Figure 3:

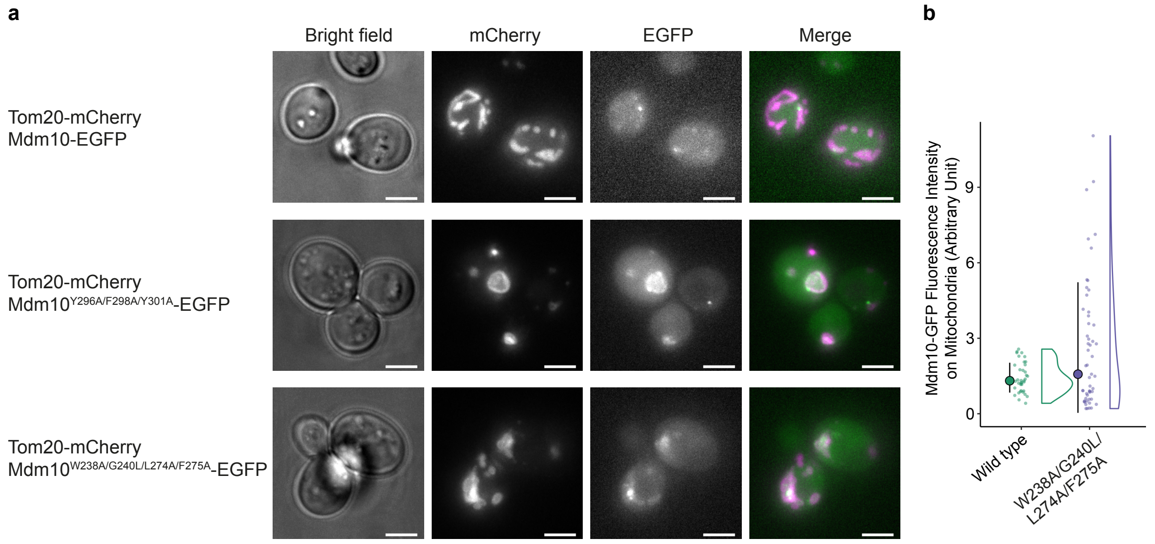

Supplementary Figure 3 legend: To verify that mutant Mdm10$^{W238A/G240L/L274A/F275A}$ is expressed and localises to mitochondria, we generated a GFP-tagged variant. **a:** Live cell imaging of yeast cells expressing Tom20-mCherry and different GFP-tagged Mdm10 variants. Similarly to wild type Mdm10 tagged with EGFP (top row) and Mdm10$^{Y296A/F298A/Y301A}$-EGFP (second row, mutation described by Ellenrieder L. *et al.*, 2016), Mdm10$^{W238A/G240L/L274A/F275A}$-EGFP (bottom row) displays mitochondrial localisation. **b:** Quantification of GFP fluorescence intensity on mitochondria, which were identified using the Tom20-mCherry signal. Each data point corresponds to one cell, large points indicate median and vertical lines MAD. Wild type: median 1.31, n=37 cells. Mdm10$^{W238A/G240L/L274A/F275A}$: median 1.57, n=53 cells. P=0.1739 (Dunn's test, one-sided). Thus, Mdm10$^{W238A/G240L/L274A/F275A}$-EGFP is present on mitochondria at similar protein levels as Mdm10-EFGP. Together these data indicate that the mitochondrial phenotype shown in Extended Data Fig. 10 is not caused by a lack of Mdm10 protein or by its mistargeting but is due specifically to the mutation of the 4 residues. Scale bars are 3 µm.

Supplementary Table 1:

System / STA map	*g_scale*	*ccc*	*voroMQA_total*	*voroMQA_inter*	*gscore*
Trimeric complex with 4 lipids / all bridges (*simulation time: 230 ns*)	**0.3**	**0.886**	**0.403**	**0.417**	**0.644**
	0.1	0.873	0.388	0.323	0.630
	0.05	0.823	0.392	0.397	0.607
	0.02	0.750	0.399	0.497	0.574
Trimeric complex with 12 lipids / all bridges (*simulation time: 180 ns*)	**0.3**	**0.887**	**0.335**	**0.333**	**0.612**
	0.1	0.883	0.328	0.277	0.605
	0.05	0.857	0.338	0.248	0.598
	0.02	0.753	0.336	0.287	0.545
Trimeric complex with Mdm10 and 4 lipids / all bridges (*simulation time: 130 ns*)	**0.3**	**0.843**	**0.382**	**0.398**	**0.612**
	0.1	0.827	0.378	0.385	0.602
	0.05	0.769	0.384	0.462	0.576
	0.02	0.714	0.371	0.442	0.543
Trimeric complex with 4 lipids / short bridges (*simulation time: 70 ns*)	0.3	0.725	0.325	0.243	0.524
	**0.1**	**0.713**	**0.340**	**0.302**	**0.526**
	0.05	0.699	0.344	0.402	0.521
	0.02	0.688	0.324	0.356	0.506
Trimeric complex with 4 lipids / long bridges (*simulation time: 70 ns*)	0.3	0.852	0.298	0.221	0.575
	**0.1**	**0.834**	**0.322**	**0.278**	**0.578**
	0.05	0.806	0.332	0.377	0.569
	0.02	0.747	0.332	0.353	0.539

Supplementary Table 1 legend: Quality assessment of MDFF-derived models (see also Extended Data Fig. 8f). The scaling factor g_scale determines the weight of the experimental STA map on the total molecular potential. ccc refers to the cross-correlation coefficient between map and model, $voroMQA_{total}$ and $voroMQA_{inter}$ refer to the global voroMQA score and the component including only inter-subunit contacts, respectively. Based on the gscore which combines the assessment parameters (see Methods), g_scale=0.3 was considered best for all but the models fit to the short and long bridges STA maps, in which cases g_scale 0.1 was considered best and was thus used for Extended Data Fig. 8g.
